# A novel machine learning-derived radiotranscriptomic signature of perivascular fat improves cardiac risk prediction using coronary CT angiography

**DOI:** 10.1093/eurheartj/ehz592

**Published:** 2019-09-03

**Authors:** Evangelos K Oikonomou, Michelle C Williams, Christos P Kotanidis, Milind Y Desai, Mohamed Marwan, Alexios S Antonopoulos, Katharine E Thomas, Sheena Thomas, Ioannis Akoumianakis, Lampson M Fan, Sujatha Kesavan, Laura Herdman, Alaa Alashi, Erika Hutt Centeno, Maria Lyasheva, Brian P Griffin, Scott D Flamm, Cheerag Shirodaria, Nikant Sabharwal, Andrew Kelion, Marc R Dweck, Edwin J R Van Beek, John Deanfield, Jemma C Hopewell, Stefan Neubauer, Keith M Channon, Stephan Achenbach, David E Newby, Charalambos Antoniades

**Affiliations:** 1 Division of Cardiovascular Medicine, Radcliffe Department of Medicine, University of Oxford, John Radcliffe Hospital, Headley Way, Oxford, UK; 2 Oxford Academic Cardiovascular CT Core Laboratory, West Wing, John Radcliffe Hospital, Headley Way, Oxford, UK; 3 British Heart Foundation Centre for Cardiovascular Science, University of Edinburgh, Chancellor's Building, 49 Little France Cres, Edinburgh, UK; 4 Edinburgh Imaging Facility QMRI, University of Edinburgh, 47 Little France Cres, Edinburgh, UK; 5 Heart and Vascular Institute, Cleveland Clinic, 9500 Euclid Avenue, Cleveland, OH, USA; 6 Department of Cardiology, Friedrich-Alexander-Universität Erlangen-Nürnberg, Ulmenweg 18, Erlangen, Germany; 7 Department of Cardiology, Oxford University Hospitals NHS Foundation Trust, John Radcliffe Hospital, Oxford, UK; 8 Caristo Diagnostics Ltd, Whichford House, Parkway Court, John Smith Dr, Oxford, UK; 9 National Centre for Cardiovascular Prevention and Outcomes, Institute of Cardiovascular Science, University College London, 1 St Martins Le Grand, London, UK; 10 Clinical Trial Service Unit, Nuffield Department of Population Health, University of Oxford, BHF Centre for Research Excellence, Big Data Institute, Old Road Campus, Roosevelt Drive, Oxford, UK; 11 British Heart Foundation Centre of Research Excellence, University of Oxford, John Radcliffe Hospital, Headley Way, Oxford, UK; 12 National Institute of Health Research Oxford Biomedical Research Centre, John Radcliffe Hospital, Headley Way, Oxford, UK

**Keywords:** Computed tomography, Adipose tissue, Radiomics, Machine learning, Risk stratification, Coronary artery disease

## Abstract

**Background:**

Coronary inflammation induces dynamic changes in the balance between water and lipid content in perivascular adipose tissue (PVAT), as captured by perivascular Fat Attenuation Index (FAI) in standard coronary CT angiography (CCTA). However, inflammation is not the only process involved in atherogenesis and we hypothesized that additional radiomic signatures of adverse fibrotic and microvascular PVAT remodelling, may further improve cardiac risk prediction.

**Methods and results:**

We present a new artificial intelligence-powered method to predict cardiac risk by analysing the radiomic profile of coronary PVAT, developed and validated in patient cohorts acquired in three different studies. In Study 1, adipose tissue biopsies were obtained from 167 patients undergoing cardiac surgery, and the expression of genes representing inflammation, fibrosis and vascularity was linked with the radiomic features extracted from tissue CT images. Adipose tissue wavelet-transformed mean attenuation (captured by FAI) was the most sensitive radiomic feature in describing tissue inflammation (*TNFA* expression), while features of radiomic texture were related to adipose tissue fibrosis (*COL1A1* expression) and vascularity (*CD31* expression). In Study 2, we analysed 1391 coronary PVAT radiomic features in 101 patients who experienced major adverse cardiac events (MACE) within 5 years of having a CCTA and 101 matched controls, training and validating a machine learning (random forest) algorithm (fat radiomic profile, FRP) to discriminate cases from controls (C-statistic 0.77 [95%CI: 0.62–0.93] in the external validation set). The coronary FRP signature was then tested in 1575 consecutive eligible participants in the SCOT-HEART trial, where it significantly improved MACE prediction beyond traditional risk stratification that included risk factors, coronary calcium score, coronary stenosis, and high-risk plaque features on CCTA (Δ[C-statistic] = 0.126, *P* < 0.001). In Study 3, FRP was significantly higher in 44 patients presenting with acute myocardial infarction compared with 44 matched controls, but unlike FAI, remained unchanged 6 months after the index event, confirming that FRP detects persistent PVAT changes not captured by FAI.

**Conclusion:**

The CCTA-based radiomic profiling of coronary artery PVAT detects perivascular structural remodelling associated with coronary artery disease, beyond inflammation. A new artificial intelligence (AI)-powered imaging biomarker (FRP) leads to a striking improvement of cardiac risk prediction over and above the current state-of-the-art.

**Figure:**
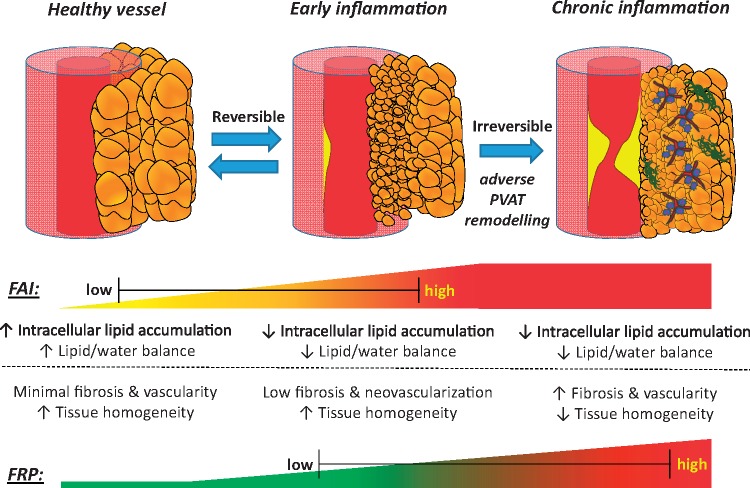

**Translational perspectives**

Perivascular adipose tissue (PVAT) changes its composition in response to coronary inflammation, as demonstrated by the perivascular Fat Attenuation Index (FAI) derived from coronary computed tomography angiography. A novel radiotranscriptomic signature of PVAT texture detects additional disease-related changes to PVAT composition, including fibrotic and microvascular remodelling. The fat radiomic profile (FRP), derived by machine learning-powered radiomic analysis of PVAT remodelling, significantly improves risk prediction for adverse cardiac events beyond the current state-of-the-art. Whereas FAI changes dynamically in response to acute coronary inflammation, FRP captures persistent structural remodelling in PVAT and provides additional risk stratification in both primary and secondary prevention.

## Introduction

Coronary artery disease (CAD) remains a leading cause of morbidity and mortality despite advances in primary and secondary prevention strategies.^1^ Coronary CT angiography (CCTA) is now a first line investigation for the assessment of possible CAD.^2–4^ The CCTA traditionally relies on the detection of obstructive lesions or coronary calcification to guide cardiovascular risk stratification and clinical decision-making.^5^^,^^6^ However, optimal medical therapy and clinical risk factor management do not always prevent acute coronary syndromes, resulting in the concept of ‘residual cardiovascular risk’, a major factor driving uncertainty in risk stratification and targeting of healthcare interventions.^7^^,^^8^ Vascular inflammation, in particular, is recognized as a major contributor to both atherosclerotic plaque formation and destabilization.^7^^,^^8^ However, conventional tests such as circulating inflammatory biomarkers are not specific enough to identify coronary inflammation, and advanced imaging tests (e.g. positron emission tomography-CT using 18F-NaF)^9^^,^^10^ are costly and not widely available, limiting their current use in clinical practice.^11^^,^^12^

It is now established that the coronary artery wall and its perivascular adipose tissue (PVAT) interact in a bidirectional manner.^12–15^ We have recently demonstrated that, in the presence of vascular inflammation, the release of pro-inflammatory molecules from the diseased vascular wall inhibits differentiation and lipid accumulation in PVAT pre-adipocytes, creating a gradient from a lipid-rich and less aqueous phase close to a non-diseased artery to lipid-poor and more aqueous phase close to an inflamed artery.^12^ In humans, this inflammation-induced gradient of PVAT composition leads to an increase in the CT attenuation from more negative [closer to −190 Hounsfield Units (HU)] to less negative (closer to −30 HU) values, as captured by the recently described perivascular Fat Attenuation Index (FAI).^12^ The FAI is a sensitive and dynamic biomarker of coronary inflammation^12^ and is a strong and independent predictor of adverse cardiac events.^16^ Furthermore, in the CRISP-CT (Cardiovascular RIsk Prediction using Computed Tomography) study,^16^ the prognostic value of FAI was reduced among patients who initiated treatment with aspirin and statin therapy after CCTA, suggesting that the risk identified by this biomarker may be modifiable.

However, chronic atherosclerosis and vascular inflammation may also be associated with structural changes in the adjacent adipose tissue, including fibrosis and microvascular remodelling.^17^^,^^18^ An imaging biomarker detecting these changes in PVAT composition could have important clinical implications for CAD diagnosis and treatment by supplementing the inflammatory burden detected by FAI. Advances in computational radiomic approaches and machine learning now enable the extraction of large amounts of quantitative information from imaging data using specific data-characterization algorithms, thus identifying imaging patterns of significant clinical value that cannot be recognized by a human reader.^19^ Linking such imaging patterns to underlying tissue biology and gene expression status in a ‘radiotranscriptomic’ approach could lead to a more individualized assessment of disease activity in patients undergoing CCTA, and could provide new biological insights in to disease mechanisms.

We hypothesized that comprehensive CCTA-based radiomic phenotyping of coronary PVAT may reveal new biomarkers of adverse remodelling in response to coronary inflammation and atherosclerosis.

## Methods

### Study design

**Figure 1:**
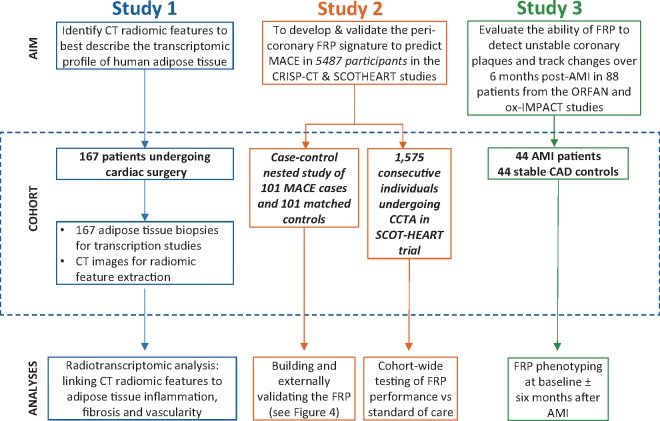
Workflow diagram. Study 1 included 167 adipose tissue biopsies from cardiac surgery patients in order to evaluate the correlation between fat biology and radiomic features. Study 2 utilized a pool of 5487 patients with coronary CT angiography who participated either in the Cardiovascular RISk Prediction using CT study or the SCOT-HEART trial, so as to develop and validate FRP, and finally to test its cohort-wide performance against the standard of care. Finally, in Study 3, we performed further external biological validation of FRP as follows: 88 patients from the Ox-IMPACT and ORFAN studies were included to test FRP’s ability to identify acute myocardial infarction-related perivascular changes and compare its ability to track longitudinal perivascular changes over a period of 6 months with that of Fat Attenuation Index. AMI, acute myocardial infarction; CAD, coronary artery disease; CRISP-CT, Cardiovascular RISk Prediction using CT study; FRP, fat radiomic profile; ORFAN, Oxford Risk Factors and Non-invasive Imaging study; Ox-IMPACT, Oxford Imaging of Perivascular Adipose tissue using Computed Tomography study; SCOT-HEART, Scottish COmputed Tomography of the HEART study.

The patient demographics and relevant clinical characteristics for each study are presented in the Supplementary material online, *Tables S1–S4*). A comprehensive workflow diagram of each study design is presented in *Figure 1*. All studies complied with the Declaration of Helsinki and were approved by local ethics committees. Informed consent was obtained from all participants.

#### Study 1

To understand how fibrosis and vascularity of adipose tissue are visualized using CT, 167 patients scheduled to undergo cardiac surgery underwent CCTA and biopsies of adipose tissue were obtained during surgery. The CT radiomic profile of adipose composition was quantified and, using a radiotranscriptomic approach, was subsequently linked to the expression of genes characterizing inflammation (e.g. tumour necrosis factor α, *TNFA*),^12^^,^^20^ fibrosis (e.g. *COL1A1*),^21^ and vascularity [e.g. endothelial-marker platelet and endothelial cell adhesion marker *CD31* (*PECAM1*)]^22^ within the visualized tissue. The study was approved by the local Research Ethics Committee (Oxford REC C 11/SC/0140).

#### Study 2

To train and subsequently validate a radiomic signature of global coronary PVAT that identifies individuals at increased risk for major adverse cardiac events (MACE), and compare it with the radiotranscriptomic signatures of inflammation, fibrosis and vascularity, we designed a case-control study of patients with a MACE (defined as the composite endpoint of cardiac mortality and non-fatal myocardial infarction, *n* = 101) within 5 years of a CCTA scan vs. controls who had no cardiac event during the same follow-up period after a CCTA [matched 1:1 for age, gender, risk factors, scanner, location (cohort) and acquisition settings (kVp)]. Both cases and controls were retrieved from the pool of 5487 patients with CCTA who participated either in the Cardiovascular RISk Prediction with CT (CRISP-CT) study^16^ or the Scottish COmputed Tomography of the HEART (SCOT-HEART) trial.^23^^,^^24^ The study design, collection of demographic data, clinical information, and outcomes for both cohorts have been previously described (Supplementary material online).^16^^,^^23^^,^^24^ The pooling of CCTA scans from multiple imaging centres with variable hardware and CCTA protocols (see Supplementary material online) was performed to facilitate the discovery of radiomic patterns that are less likely to be confounded by scanner- or centre-specific variables and are more likely to be generalizable and valid across different centres and CT scanner types. The selected scans were then randomly split into a training//internal validation (80%) and an external validation set (20%) to train and validate a high-risk PVAT radiomic signature (fat radiomic profile, FRP) that can discriminate high-risk MACE from low-risk non-MACE cases.

Once this signature (FRP) was trained and tested in the external validation set, its *cohort-wide* performance for detection of residual cardiac risk was explored in 1575 consecutive eligible patients from the SCOT-HEART trial (SCOT-HEART, NCT01149590), a population in which CCTA [as well as non-contrast cardiac CT for Agatston coronary calcium scoring (CCS)] was used to guide changes in clinical care. The aim was to explore whether this radiomic signature could add value in cardiac risk prediction over and above traditional risk factors and detect a residual risk not modified by subsequent interventions (Supplementary material online). The median follow-up period was 4.8 years (interquartile range: 4.2–5.7 years), and adverse cardiac events, including death, non-fatal acute myocardial infarction (AMI) and revascularization events were recorded as described previously.^23^^,^^24^

#### Study 3

To compare the ability of FRP to track perivascular changes related to AMI in comparison to the inflammation-based FAI-index, a total of 44 AMI patients underwent CCTA within 96 h of presentation ± follow-up CCTA after six event-free months, as part of a prospective study aiming to assess the association between vulnerable plaques and pericoronary fat radiomic features [Oxford Imaging of Perivascular Adipose tissue using Computed Tomography (Ox-IMPACT) study, Research Ethics Committee Reference 17/SC/0058].^25^^,^^26^ A total of 44 matched patients with stable CAD were selected as the control group [Oxford Risk Factors and non-invasive Imaging (ORFAN) study; Research Ethics Committee Reference 15/SC/0545].

### Coronary CT angiography protocol, data collection and definitions

The detailed CCTA protocols for image acquisition are presented in the Supplementary material online and in previously published studies.^12^^,^^16^^,^^23^^,^^24^ Coronary artery stenosis assessment and the presence of high-risk plaque (HRP) features (defined as the presence of at least one of the following: low-attenuation plaque, positive remodelling, napkin-ring sign or spotty calcification) were performed as previously described (Supplementary material online).^27^^,^^28^

### Radiomic analysis

#### Image transfer and segmentation

All images were first anonymized locally and subsequently transferred to the Oxford Academic Cardiovascular Computed Tomography (OXACCT) core lab (Oxford, UK) for analysis on a dedicated workstation by investigators blinded to population demographics and outcomes. All analyses were performed using the standard operating procedures of the OXACCT core lab and for FRP measurements both intra-observer [intra-class correlation coefficient (ICC) 0.995 (95% CI: 0.982–0.999), *P* < 0.001] and inter-observer agreement amongst three independent operators [ICC 0.938 (95% CI: 0.861–0.977), *P* < 0.001] was excellent. Subcutaneous adipose tissue (Study 1) was segmented on three consecutive axial slices at the caudal end of the sternum (xiphoid process) by selecting all subcutaneous voxels in the −190 to −30 HU range. Coronary PVAT (Studies 2 and 3) was defined as all voxels in the HU range of −190 to −30 HU located within a radial distance from the outer vessel wall equal to the diameter of the respective vessel, as previously described.^16^ Perivascular adipose tissue segmentation was performed around the proximal to distal right coronary artery (RCA; anatomical segments #1, 2 and 3 based on the Society of Cardiovascular CT anatomical classification) and the combined left main and proximal to mid left anterior descending artery (LCA: left coronary artery; anatomical segments #5, 6, 7).^29^^,^^30^ The left circumflex artery was not analysed given its variable anatomy and small calibre, as already described in the CRISP-CT study.^16^

#### Radiomic characterization

Calculation of radiomic features was performed on contrast CCTA scans using 3D Slicer (v.4.9.0-2017-12-18 r26813).^31^ For each unique AT segmentation, a total of 843 radiomic features were calculated, ranging from shape-related to first- and higher-order (texture) statistics (Supplementary material online, *Tables S5–S12*), using the SlicerRadiomics extension which incorporates the Pyradiomics library into 3D Slicer.^32^ More details on segmentation, bin discretization, radiomic matrix symmetry, and wavelet transformations can be found in the Supplementary material online. Fat radiomic profile was independent of the mean arterial lumen attenuation as measured at the aortic root (Rho = −0.005, *P* = 0.90), as the segmented PVAT volumes is sufficiently (>2 voxels) away from the outer wall of the coronary artery, thus the measurements are not affected by the lumen partial voluming effect. Moreover, texture radiomic phenotyping assesses the relative rather than absolute changes in PVAT attenuation, explaining why the measurements are independent of the absolute, baseline attenuation. Epicardial adiposity and perivascular FAI (as validated in our previous work^12^^,^^16^) were also quantified using dedicated software (Aquarius Workstation^®^ V.4.4.11-13, TeraRecon Inc., Foster City, CA, USA for basic segmentation and CaRi-HEART proprietary algorithms (Caristo Diagnostics Ltd, Oxford, UK) for final calculation), as described previously.^16^

### Adipose tissue collection, ribonucleic acid isolation, and gene expression studies

In Study 1, adipose tissue biopsies were harvested from the incision site in 167 patients undergoing cardiac surgery and transferred to the lab on ice, as previously described.^12^ Total ribonucleic acid (RNA) was isolated by phenol:chloroform (1:5 ratio) separation followed by magnetic beads-based RNA purification (using a KingFischer magnetic particle robotic processor). RNA was reverse-transcribed to complementary deoxyribonucleic acid (cDNA) by using SuperScript VILO mastermix. Quantitative real-time polymerase chain reactions (PCR) for *TNFA*, *COL1A1*, *CD31* and *PPIA* (housekeeping gene), were performed by using TaqMan probes on a QuantStudio 7 flex real-time PCR system (details in the Supplementary material online).

### Statistical analysis

Participant demographics are summarized as numbers (percentages) or median (25th to 75th percentile) for categorical and continuous variables, respectively (unless specified otherwise). Between-group comparisons were performed using Pearson’s χ^2^ or Fisher’s exact test for categorical variables, and Mann–Whitney’s *U* test for continuous variables.

In Study 1, the strength of association between attenuation- and texture-based radiomic features of AT (*n* = 825 after excluding 18 shape- and volume-related features) and the relative gene expression of *TNFA*, *COL1A1*, and *CD31* in respective adipose tissue biopsies was summarized using Manhattan plots [−log_10_(*P-*values) of the Spearman’s rho coefficients]. Bonferroni correction was applied by dividing the significance level of *α* = 0.05 by the number of components that described 99.5% of the radiomic variation, as described elsewhere.^33^ In order to assess the incremental value of texture analysis for description of adipose tissue biology, stepwise multivariable linear regression models were built with gene expression as the dependent variable and age, sex, body mass index, hypertension, dyslipidaemia, and diabetes mellitus (block 1), followed by the addition of mean attenuation (block 2), and the three top principal components of the AT ‘radiome’ (block 3, *F*-level cut-off of 0.05 and 0.10 for inclusion/exclusion in the model). Gene expression profiles were log-transformed prior to inclusion in the models. The *F*-metric and *F*-test were used to assess the improvement in model performance at each step.

In Study 2, only features with an inter-observer ICC ≥0.9 were included in all subsequent analyses (*n* = 15 scans analysed by two independent analysts). Correlation plots (using the non-parametric Spearman’s rank correlation coefficient) and a heatmap depicting the variance of the *Z*-score-transformed individual radiomic features across the SCOT-HEART population are graphically presented. Further, hierarchical clustering of the individual radiomic features was performed using the squared Euclidean distance and the Ward method.

To address the multi-dimensionality and possible redundancy of the radiomic dataset, the amount of pairwise correlations was reduced at the level of |rho|≥0.9 using the *findCorrelation* function of the *caret* package in *R*. In an initial assessment of the dataset, a Manhattan plot was created based on the value of each radiomic feature in discriminating the MACE from the non-MACE cases [−(log_10_) of the *P*-value from univariable receiver operating characteristic (ROC) curve analysis]. Subsequently, the nested case-control study in Study 2 was randomly split into a training/internal validation (80%) and external validation set (20%), ensuring an equal representation of events and non-events in both splits. A random forest method was selected to train a model to discriminate 5 year MACE from no MACE, since it enables modelling of non-linear relationships, can train on small datasets, is less sensitive to outliers and has been previously used in patient-specific predictive modelling.^34^ Further details on centring, scaling of radiomic features, recursive feature elimination, internal cross-validation, and final validation can be found in the Supplementary material online. The final product of the random forest algorithm (namely the probability of belonging to the MACE vs. no MACE group) was defined as the FRP. The biological meaning of the most important constituent features of FRP was assessed by presenting the significance level of association with adipose tissue fibrosis (*COL1A1* relative expression), inflammation (*TNFA* relative expression), and vascularity (*CD31* relative expression), as assessed in Study 1.

When applied in a cohort-wide setting in the SCOT-HEART trial, an optimal FRP cut-off point was selected to define high- vs. low-risk groups by identifying the value that maximizes the log-rank statistic for MACE. Its prognostic value for (i) MACE; (ii) a composite endpoint of MACE, and late revascularization (revascularization ≥6 weeks post-CCTA; corresponding to the timepoint for assessment of the certainty of coronary heart disease diagnosis in the SCOT-HEART trial),^23^ and (iii) non-cardiac mortality was then assessed in both univariable Kaplan–Meier curves and Cox regression models adjusted for age, sex, systolic blood pressure, diabetes mellitus, body mass index, smoking status, presence of CAD (≥50% stenosis), total cholesterol and high-density lipoprotein levels as well as scanner type, presence of HRP features and Agatston CCS [log(CCS + 1); details in the Supplementary material online]. The incremental value of FRP for prediction of MACE beyond a baseline model consisting of the aforementioned factors was assessed by comparing the time-dependent ROC curves of the two nested models (traditional risk factors ± FRP) at 5 years post-CCTA.

In Study 3, the distribution of FRP between AMI and matched stable CAD patients was compared using the non-parametric Mann–Whitney *U* test. The changes in FAI or FRP between baseline and follow-up were assessed using a paired, non-parametric Wilcoxon-signed rank test. On the other hand, changes in FAI along a culprit lesion at baseline (within 96 h after AMI) and at 6 months were compared with a repeated measures two-way analysis of variance test with timepoint × length interaction.

Statistical analyses were performed in the R environment (R version 3.4 and R Studio version 1.1.453) and using IBM SPSS Statistics for Windows (Version 22.0. IBM Corp. Released 2013. Armonk, NY, USA). All tests were two-sided and *α* was set at 0.05, unless specified otherwise.

## Results

### Adipose tissue radiomic characteristics for detection of tissue inflammation, fibrosis and vascularity (Study 1)

**Figure 2:**
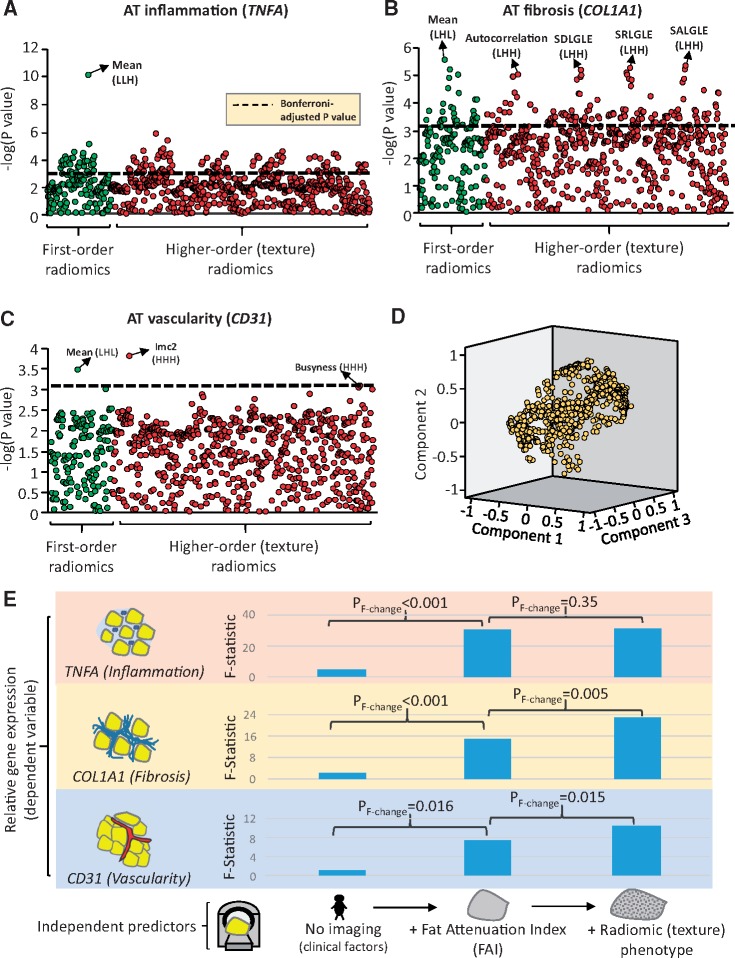
Radiomic phenotyping to detect biological hallmarks of dysfunctional adipose tissue. (*A–C*) Manhattan plots presenting the strength of association [−log_10_(*P*-value) of Spearman’s rho] between adipose tissue radiomic features and the relative gene expression of *TNFA* (inflammation), *COL1A1* (fibrosis), and *CD31* (endothelial marker, vascularity)*. Radiomic features were split into two groups, consisting of first-order statistics (green colour, derived from simple attenuation histogram analysis) or higher-order statistics (red colour, reflecting the radiomic texture and spatial interrelation of voxels). The dotted line represents the Bonferroni-adjusted significance level (*α* = 0.00068). (*D*) Component plot of the three principal components of the adipose tissue radiome. (*E*) Comparison of nested linear regression models with relative gene expression as the dependent variable and (i) clinical risk factors alone (Model 1: age, sex, hypertension, hypercholesterolaemia, diabetes mellitus, body mass index); (ii) Model 1 + Mean Attenuation (Model 2); and (iii) Model 2 + PVAT radiome (first three principal components) as the independent predictors. The *F* statistic at each step is presented and compared with the previous step using the *F-*test. Imc, informational measure of correlation 2; L/H, low/high wavelet transformation; SALGLE, small area low grey level emphasis; SDLGLE, small dependence low grey level emphasis; SRHGLE, Short Run Low Grey Level Emphasis. *Relative gene expression was calculated using cyclophilin A (*PPIA*) as the housekeeping gene.

Radiotranscriptomic analysis of human adipose tissue revealed that wavelet-transformed mean attenuation (a first-order statistic which forms the basis of the FAI) was the best-performing metric for detection of adipose tissue inflammation, as assessed by the relative expression of *TNFA* (*Figure 2A*). However, higher-order statistics reflecting the radiomic texture of adipose tissue on CT imaging had comparable or even higher accuracy than mean attenuation (*Figure 2B, C*) in detecting fibrosis and vascularity, as assessed by the relative expression of *COL1A1* (collagen type 1 alpha 1 chain) and the endothelial-marker *CD31* (platelet-endothelial cell adhesion molecule-1). In multivariable analysis, inclusion of the three principal components of the adipose tissue ‘radiome’ (*Figure 2D*) improved the predictive value of a model including clinical risk factors and mean attenuation to detect adipose tissue fibrosis and vascularity, but not inflammation (*Figure 2E*). This proof-of-concept analysis suggests that texture phenotyping of adipose tissue provides a non-invasive means to detect fibrosis and adipose tissue microvascular remodelling.

### Searching for a radiomic signature in perivascular adipose tissue that predicts cardiac risk beyond attenuation (Study 2a)

**Figure 3:**
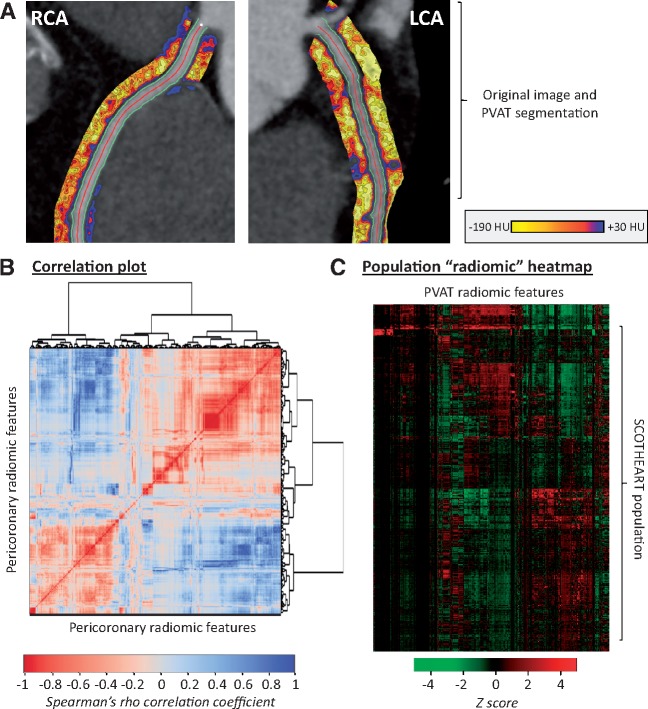
Radiomic phenotyping of coronary perivascular adipose tissue. (*A*) The perivascular adipose tissue of the right and left coronary arteries (left main and proximal to mid left anterior descending artery) was segmented and used to calculate a number of shape-, attenuation-, and texture-related statistics. (*B*) Correlation plot of all 1391 stable radiomic features in the SCOT-HEART population (*n* = 1575 patients), with hierarchical clustering revealing distinct clusters of radiomic variance. (*C*) Heatmap of scaled radiomic features in the SCOT-HEART population revealing between-patient variance across the cohort. LCA, left coronary artery; PVAT, perivascular adipose tissue; RCA, right coronary artery.

Radiomic phenotyping of coronary PVAT around the RCA and LCA produced a total of 1686 radiomic features (843 features around each vessel; Supplementary material online, *Figure S1* and *Figure 2A*). Stability analysis identified 1391 (82.0%) features with an ICC ≥ 0.9 (Supplementary material online, *Figure S2*). A correlation plot with further hierarchical clustering of these stable 1391 radiomic features in 1575 SCOT-HEART patients revealed distinct clusters of highly correlated features (*Figure 3B*), with notable variance across the cohort population (*Figure 3C*).

**Figure 4:**
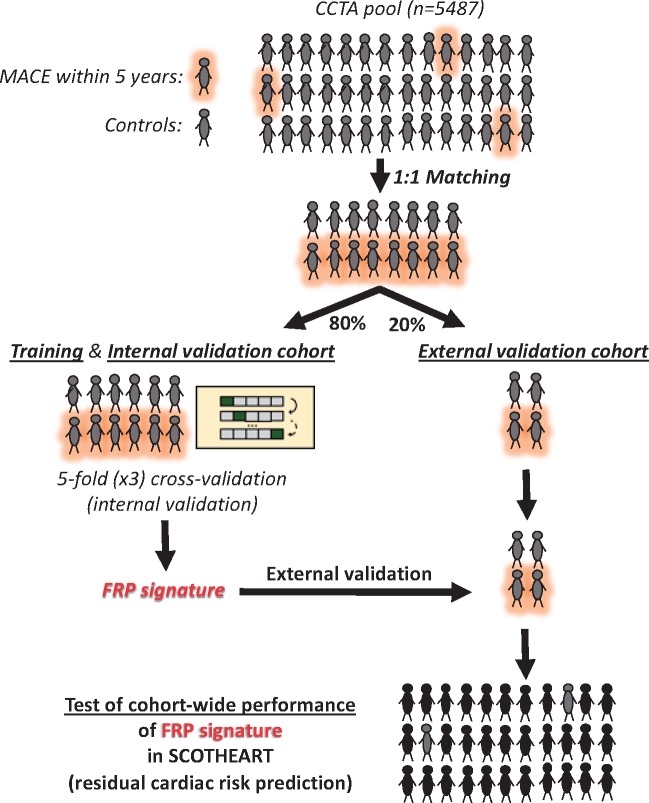
Identifying the high-risk pericoronary fat radiomic profile (Study 2). Cases of individuals that suffered a major adverse cardiac events (cardiac death or non-fatal myocardial infarction) within 5 years of their coronary CT angiography scan (*n* = 101) and matched controls (*n* = 101) were selected from a pool of 5487 individual coronary CT angiography scans with follow-up for outcomes. These were randomly split into a training and internal validation set (80% of observations and events, using repeated five-fold cross-validation) and an external validation set (the remaining 20%) to train and test a random forest model to discriminate MACE from non-MACE cases. The product of the random forest model was defined as the fat radiomic profile. The FRP was subsequently measured in 1575 consecutive eligible cases from the SCOT-HEART study to assess its cohort-wide performance in predicting the residual cardiac risk among individuals undergoing clinically indicated coronary CT angiography. CCTA, coronary computed tomography angiography; FRP, fat radiomic profile; MACE, major adverse cardiac events.

**Figure 5:**
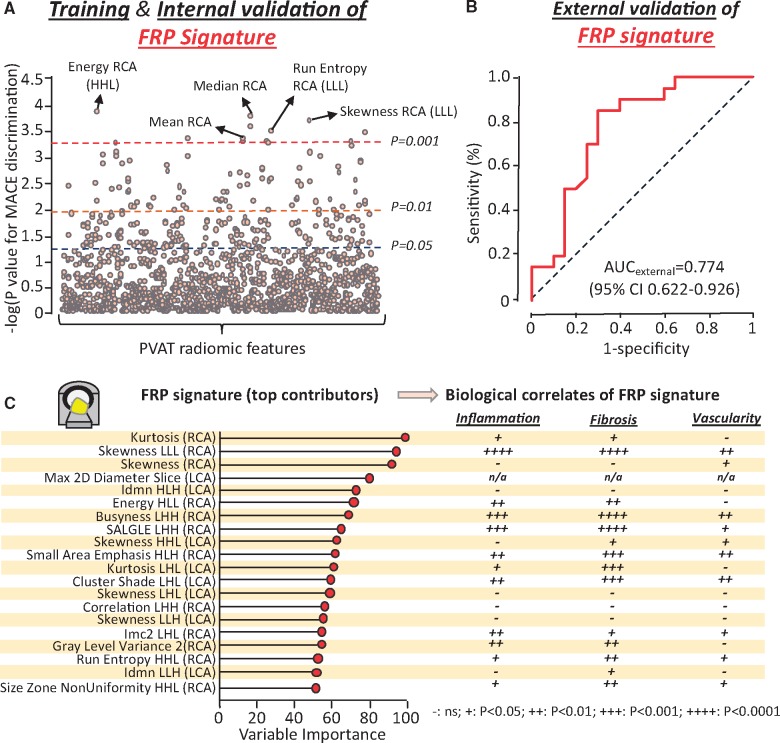
The pericoronary fat radiomic profile. (*A*) A forest plot of the discriminatory value of each radiomic feature in univariable analysis. (*B*) Validation of the final model in the validation set (20% of the initial sample). (*C*) Variable importance of the top 20 radiomic features of the final random forest model and corresponding strength of association with adipose tissue inflammation, fibrosis and vascularity, as assessed in Study 1 (^++++^*P* < 0.0001; ^+++^*P* < 0.001. ^++^*P* < 0.01, ^+^*P* < 0.05, and ^–^*P* ≥ 0.05). AUC, area under the curve; CI, confidence interval; CCTA, coronary computed tomography angiography; FRP, fat radiomic profile; H, high filter; Imc2, informational measure of correlation 2; Idmn, inverse difference moment normalized; L, low filter; LCA, left coronary artery; MACE, major adverse cardiac events; MI, myocardial infarction; RCA, right coronary artery.

A total of 101 MACE cases (within 5 years of CCTA) and 101 matched controls with MACE-free 5 years of follow-up (Supplementary material online, *Table S2*) were retrieved from the discovery CCTA pool and used in a case-control study to identify the presence of a distinct radiomic signature that is independently associated with cardiac risk (*Figure 4*). The two groups were well-matched for age, traditional cardiovascular risk factors, systemic or epicardial obesity and CT scanning parameters (Supplementary material online, *Table S2*). In univariable ROC analysis, a total of 22 (1.6%), 86 (6.2%) and 241 (17.3%) of the 1391 stable radiomic features were found to discriminate MACE from non-MACE cases (*P* < 0.001, *P* < 0.01, and *P* < 0.05, respectively; *Figure 5A*).

In order to identify a single radiomic signature that combines the diagnostic value of all these features, the study population was randomly split into a training/internal validation (80%) and an external validation set (20%; *Figure 4*). Reduction of pairwise correlations at the level of |Spearman’s rho|≥0.9 resulted in 335 independent features. Recursive feature elimination with a random forest algorithm and repeated five-fold cross-validation showed a plateau in the accuracy of the trained model with a selection of 64 features (Supplementary material online, *Figure S3*). Following three rounds of repeated five-fold cross-validation in order to validate the best-performing model internally and avoid overfitting, the discriminatory value of the model (FRP) was confirmed in the remaining validation set [AUC 0.774 (95% CI 0.622–0.926)] (*Figure 5B* an*d*Supplementary material online, *Figure S4*).

To understand the biological interpretation of FRP, we then searched for the overlap between the radiotranscriptomic signatures for adipose tissue inflammation, fibrosis and vascularity and the features included in the FRP (*Figure 5C*). Notably, FRP consists of a range of PVAT radiomic features that are differentially associated with either inflammation, fibrosis and vascularity or any combination of these hallmarks of dysfunctional adipose tissue.

### Exploring the prognostic value of the fat radiomic profile in SCOT-HEART (Study 2b)

**Figure 6:**
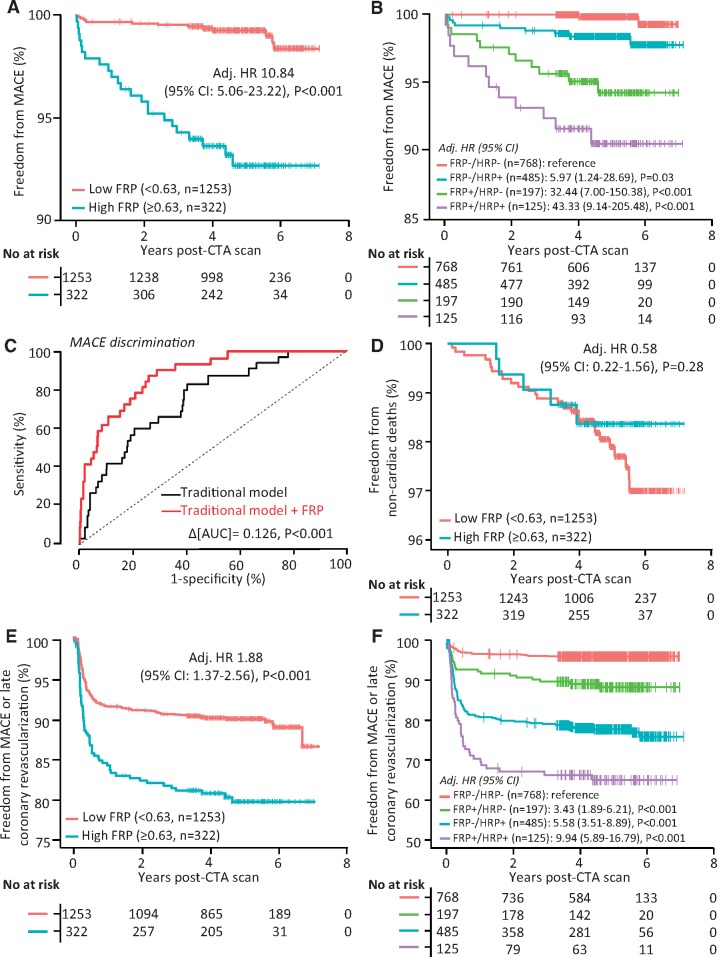
Prognostic value of the pericoronary fat radiomic profile. (*A*, *B*) Kaplan–Meier curves and adjusted hazard ratios for major adverse cardiac events across strata of fat radiomic profile [≥0.63 (FRP+) vs. 0.63 (FRP−)] and high-risk plaque features. (*C*) Time-dependent receiver operating characteristic curves (*t* = 5 years post-coronary CT angiography) for two nested prediction models consisting of age, sex, systolic blood pressure, diabetes mellitus, body mass index, smoking status, presence of coronary artery disease (≥50% stenosis), total cholesterol, high-density lipoprotein levels, scanner type, presence of high-risk plaque, as well as Agatston coronary calcium scoring [log(CCS + 1)] with (AUC: 0.880) or without (AUC: 0.754) FRP. (*D*) Kaplan–Meier curves and adjusted hazard ratios for non-cardiac mortality, as well as a composite endpoint of major adverse cardiac events and/or late revascularization (*E*, *F*) across strata of FRP and high-risk plaque features. AUC, area under the curve; CI, confidence interval; CCS, coronary calcium score; CCTA, coronary computed tomography angiography; FRP, fat radiomic profile; HR, hazard ratio; HRP, high-risk plaque feature; MACE, major adverse cardiac events.

We next determined the FRP signature in the 1575 eligible CCTA scans from the SCOT-HEART study. Over a median follow-up period of 4.8 (25th to 75th percentile: 4.2–5.7) years, one confirmed cardiac death, 33 non-fatal AMI events, 176 late revascularization events and 32 non-cardiac death events were recorded (Supplementary material online, *Table S3*). FRP was positively associated with the adjusted risk of MACE [per 0.01 increments: adj. HR 1.12 (95% CI 1.08–1.15), *P* < 0.001]. For an optimal cut-off point of 0.63 (Supplementary material online, *Figure S5*) individuals with FRP ≥ vs. <0.63 (FRP+ vs. FRP−) had a 10.8-fold higher risk of MACE, after adjustment for age, sex, SBP, total cholesterol, HDL, diabetes mellitus, smoking, BMI, obstructive disease, scanner type, presence of HRP features, and calcium score (*Figure 6A*). Of note, FRP was not related to the presence of HRP features (Rho = 0.004, *P* = 0.87), and correlated very weakly with calcium score (rho = 0.07, *P* = 0.007). FRP+/HRP+ individuals had a 43-fold higher risk of MACE compared with the FRP−/HRP− participants, after adjustment for age, sex, SBP, total cholesterol, HDL, diabetes mellitus, smoking, BMI, and scanner type (*Figure 6B*). FRP significantly improved the predictive value of a traditional model consisting of age, sex, systolic blood pressure, diabetes mellitus, body mass index, smoking status, presence of CAD (≥50% stenosis), total cholesterol, high-density lipoprotein levels, scanner type, presence of HRP, as well as Agatston CCS [log(CCS + 1); *Figure 6C*]. In further analyses, FRP did not predict non-cardiac mortality (*Figure 6D*) but remained an independent predictor of a composite endpoint of MACE or late coronary revascularization (≥6 weeks post-CCTA; *Figure 6E, F*) confirming the cardiac-specific nature of the biomarker.

### Pericoronary fat radiomic profile in acute myocardial infarction (Study 3)

**Figure 7:**
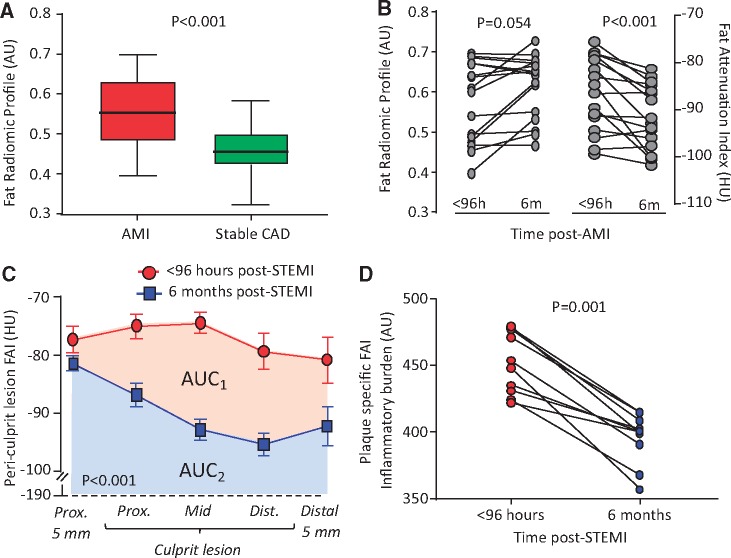
Pericoronary fat radiomic profile in acute myocardial infarction. (*A*) Tukey box-plot of FRP values in patients scanned within 96 h of acute myocardial infarction and matched controls undergoing clinical coronary CT angiography for suspected stable coronary artery disease (*n* = 44 per group). (*B*) FRP and perivascular Fat Attenuation Index in acute myocardial infarction patients scanned within 96 hours of admission and 6 months later (*n* = 16 per group). (*C*) In a subgroup of patients with ST-elevation myocardial infarction (STEMI) at baseline, Fat Attenuation Index changes were particularly pronounced in the region directly adjacent to the culprit lesion (*n* = 10). (*D*) Change in Fat Attenuation Index-associated inflammatory burden, defined as the area under the curve of Fat Attenuation Index measured along a given culprit lesion within 96 h of an acute ST-segment elevation myocardial infarction (STEMI) and 6 months later. AMI, acute myocardial infarction; AU, arbitrary units; CAD, coronary artery disease; FAI, Fat Attenuation Index; HU, Hounsfield Units; STEMI, ST-segment elevation myocardial infarction. *P*-values derived from Mann–Whitney *U* test (*A*), Wilcoxon-signed rank test (*B*, *D*) and two-way repeated measures analysis of variance with timepoint/distance interaction (*C*).

Radiomic phenotyping of 44 AMI patients scanned within 96 hours of admission and 44 matched controls undergoing clinical CCTA for stable CAD revealed higher FRP values (consistent with adverse PVAT remodelling) in the AMI vs. the control group (*Figure 7A*), despite no differences in demographics, clinical risk factors or medications (Supplementary material online, *Table S4*).

Finally, in a subgroup of 16 patients who underwent sequential CCTA scanning within 96 hours of admission for AMI and 6 months later, no significant changes were observed in FRP. In contrast, perivascular FAI around the RCA, a specific biomarker of the overall coronary inflammation, changed dynamically at 6 months (*Figure 7B*). In a subgroup of 10 patients with STEMI, these changes were particularly pronounced in the region directly adjacent to the culprit lesion, demonstrating significant changes in local FAI and the FAI-defined plaque inflammatory burden at 6 months (*Figure 7C, D*). These findings further support the notion that FAI flags reversible, dynamic changes in PVAT composition (e.g. adipocyte size and lipid accumulation), whereas FRP complements this information with detection of more advanced, irreversible changes, such as adipose tissue vascularity and fibrosis.

## Discussion

The recent development of the perivascular FAI, a CCTA-derived biomarker of coronary inflammation based on quantification of PVAT attenuation shifts, highlights the potential for complementary imaging biomarkers that can describe the full phenotypic heterogeneity of PVAT composition and remodelling and its clinical importance. Using a radiotranscriptomic approach, we now demonstrate that specific texture patterns within the radiomic profile of PVAT describe fibrosis and vascularity, reflecting permanent changes in adipose tissue induced by chronic coronary inflammation.^17^ Following this discovery, we used machine learning to build a new radiomic signature of high-risk PVAT around the coronary arteries, namely the perivascular FRP, that relies on detection of such changes to identify individuals at increased cardiac risk. Application of this method in the SCOT-HEART trial revealed an association between adverse coronary PVAT remodelling captured by FRP and residual cardiac risk. Contrary to FAI, which changes dynamically and can be used to prospectively monitor responsiveness to risk reduction measures, FRP detects persistent adverse structural remodelling in PVAT and an associated residual risk not confounded by concomitant medications or other acute processes (*Take home figure*).

**Take home figure:**
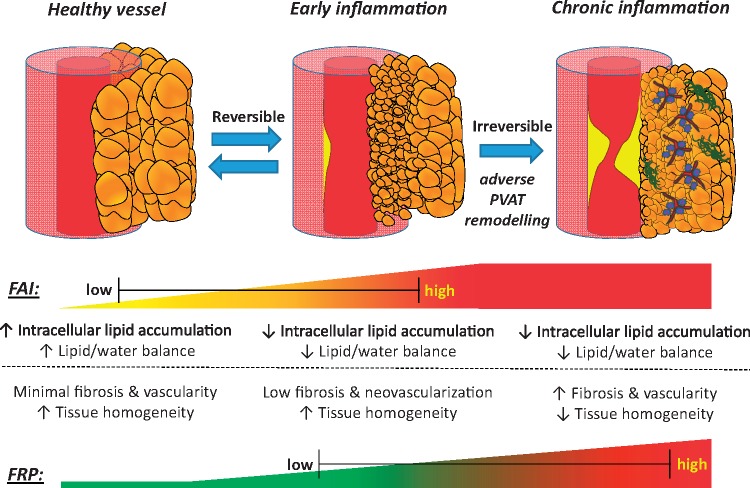
Fat radiomic profile as a marker of adverse perivascular adipose tissue remodelling. Coronary inflammation is associated with phenotypic changes in perivascular adipose tissue, characterized by decreased adipocyte size and intracellular lipid accumulation. This phenotypic shift forms the basis of the CT-derived Fat Attenuation Index that characterizes attenuation changes in perivascular adipose tissue. However, chronic vascular inflammation and atherosclerotic disease are associated with further, irreversible changes in perivascular adipose tissue composition, such as increased extracellular fibrosis and microvascular remodelling. Those changes can now be detected by analysing the radiomic phenotype of perivascular adipose tissue on coronary CT angiography imaging. A comprehensive analysis of volumetric, attenuation-based and texture-based metrics of coronary perivascular adipose tissue on coronary CT angiography imaging carries incremental prognostic value in cardiac risk prediction and highlights the critical role of perivascular adipose tissue in human atherosclerotic cardiovascular disease. CCTA, coronary computed tomography angiography; FAI, Fat Attenuation Index; FRP, fat radiomic profile; PVAT, perivascular adipose tissue.

### Perivascular adipose tissue as a sensor of vascular biology

We have recently discovered that vascular inflammation drives changes in PVAT, due to inside-to-outside signals released from the vascular wall.^12–15^ A new imaging biomarker, the perivascular FAI, detects vascular inflammation by capturing spatial shifts in PVAT composition and lipid content as CT attenuation gradients around the coronary arteries.^12^ The FAI was recently validated in the CRISP-CT study,^16^ where it was found to have prognostic value for all-cause and cardiac-related mortality. However, FAI appeared to lose its prognostic value in a sub-population of the CRISP-CT study which started treatment with statins and aspirin post-CCTA, suggesting that the risk identified by FAI may be modifiable.^16^ This led to the need for additional biomarkers that would detect permanent changes in PVAT composition that would be less affected by treatments targeting vascular inflammation.

### Discovering novel radiotranscriptomic signatures of adverse perivascular adipose tissue remodelling

Imaging studies in oncology have shown that specific shape- and texture-related patterns generate tumour phenotypes that are independently linked to the underlying tumour biology and to clinical prognosis.^35^ Such patterns, often derived from complex mathematic formulae, are invisible to the naked eye of experienced radiologists and clinicians.^36^ Radiomic approaches map these qualitative imaging features and generate a number of quantitative variables that describe the imaged structure. In a recent study, Kolossvary *et al*.^33^ showed that radiomic features can reliably identify HRPs with napkin-ring sign and discriminate metabolically active from inactive lesions.^37^ Radiomic analysis of adipose tissue is another novel method that requires investigation.

Further to lipolysis, reduced adipogenesis and water accumulation within PVAT, vascular inflammation also triggers more permanent changes in the perivascular space, such as fibrosis and neoangiogenesis,^17^^,^^18^ which could also be captured by CCTA. In a radiotranscriptomic experiment using adipose tissue biopsies from 167 patients undergoing cardiac surgery, we searched for radiomic features that would best describe the gene expression of *TNFA* (marker of inflammation), *COL1A1* (marker of fibrosis), and *CD31* (marker of vascularity). Indeed, as expected,^12^ features describing adipose tissue attenuation (captured by the FAI) were the most descriptive of *TNFA* expression. However, features associated with the radiomic texture of adipose tissue enabled a more accurate detection of more permanent changes in its phenotype, such as fibrosis (*COLA1* expression) and microvascular remodelling (endothelial-marker *CD31* expression) that affect the histological (and therefore radiomic) heterogeneity of adipose tissue. Of note, different radiomic patterns were differentially associated with tissue inflammation, fibrosis, and vascularity, suggesting that a comprehensive radiomic phenotyping approach is needed to accurately describe the biological variation of human adipose tissue.

### Developing and validating the high-risk fat radiomic profile

In a discovery study that included individuals who developed a MACE within 5 years from the CCTA and matched controls, we applied a machine learning-based approach with multiple rounds of validation, which enabled us to discover a high-risk PVAT radiomic profile (FRP) that is linked to increased cardiac risk. Importantly, FRP offered incremental prognostic information beyond current CCTA-based tools (including Agatston CCS, HRP features and luminal stenosis), suggesting a residual level of cardiac risk that is not detected by traditional risk stratification algorithms. This signature captured not only the attenuation features included in FAI but also features capturing fibrosis and vascularity of PVAT. To see whether this radiomic signature captures permanent changes induced by chronic inflammation in PVAT, the high-risk signature (FRP) was applied in the CCTA arm of the SCOT-HEART trial. This population had CCTA-guided management, with many risk-modifying interventions post-CCTA, thus affecting the clinical value of their baseline FAI measurement. Despite this, we observed that FRP retained its prognostic value for future MACE, with incremental value beyond the current state-of-the-art in CCTA-based risk prediction.

### Fat radiomic profile and acute myocardial infarction

Chronic inflammation leads to accelerated atherogenesis and induces plaque instability leading to acute coronary syndromes.^8^ In further analysis, FRP analysis revealed adverse PVAT remodelling in patients presenting with AMI compared with matched controls with stable disease. After performing serial CCTA scans in patients with AMI, FRP remained unchanged, whereas FAI was significantly decreased following 6 months of optimal medical therapy. These findings suggest that FRP (a patient-specific index) may be a useful biomarker for first-time screening of individuals undergoing CCTA, whereas FAI (a segment or plaque-specific index) captures the dynamic inflammatory burden of the coronary vasculature and can be used to track longitudinal changes in coronary inflammation.

### Limitations

The limited number of adverse events in the SCOT-HEART population (34 MACE events over a median follow-up of 4.8 years), which is typical of a low-risk patient cohort undergoing CCTA, means that further testing in independent cohorts may be needed to refine and calibrate the radiomic models. This will be facilitated by the development of automated software solutions which will reduce the computationally heavy, long analysis times required with current software. Currently, analysis time for full segmentation and radiome profile extraction takes ∼45 min per patient. New applications under way will use online Graphics Processing Units (GPU) in a cloud environment, automating detection of the coronary arteries and pericardium, perivascular space segmentations, feature extraction for calculation of FRP/FAI, with simultaneous corrections for all the other technical and anatomical information needed for artificial intelligence (AI)-based proprietary algorithms to perform the whole process, which is expected to last <5 min per patient. Also, due to the variable anatomy of the left circumflex coronary artery, analysis of FRP around that artery was not performed in this study. Future research in larger cohorts is needed, to allow reliable application of fat radiomic profiling around the left circumflex artery.^16^ In addition, intracoronary imaging studies^38^^,^^39^ may be needed to better characterize the association of PVAT radiome with coronary atherosclerosis phenotype, in our effort to identify the ‘unstable plaque’ and ultimately the ‘unstable patient’. Finally, a critical validation study (VIP study; Validation study for introduction of novel computed tomography Imaging biomarkers in clinical Practice, Oxford RECA/19/SC/0273) is currently underway aiming to provide the necessary adjustment factors for the interpretation of quantitative CT biomarkers across a range of hardware and scan/reconstruction settings.

## Conclusions

We present a high-risk radiomic signature of coronary PVAT, derived from machine learning-based analysis of traditional CCTA scans, which detects adverse structural changes associated with PVAT fibrosis and microvascular remodelling. FRP significantly improves risk prediction for adverse clinical events beyond the current state-of-the-art and discriminates patients with AMI from those with stable disease. Whereas FAI (attenuation) changes dynamically in response to acute coronary inflammation, FRP (radiomic texture) captures more permanent structural changes in PVAT and provides additional risk stratification. The combination of FAI (a component of FRP) and FRP facilitates the development of a more comprehensive individualized cardiac risk profile for each patient. In conclusion, radiomic characterization of PVAT by means of the FRP is a novel, promising approach to capture adverse PVAT remodelling around the coronary arteries and its associated residual cardiac risk.

## Supplementary Material

ehz592_Supplementary_Data

## Notes

Parts of this work received the Young Investigator Award (1st prize) of the European Society of Cardiology Congress (ESC) 2018, in the ‘Clinical Cardiology’ Category. The main findings of this work are presented as late-breaking abstract in the Basic and Translational Science category at the ESC Congress 2019 (Abstract #7236).

**See page 3544 for the editorial comment on this article (doi: 10.1093/eurheartj/ehz717)**
