# Supplementary material for: A novel machine learning-derived radiotranscriptomic signature of perivascular fat improves cardiac risk prediction using coronary CT angiography
Source: Eur Heart J. 2019 Sep 3;40(43):3529–43. doi: 10.1093/eurheartj/ehz592 (PMC6855141; doi:10.1093/eurheartj/ehz592)
Supplement: ehz592_Supplementary_Data [file ehz592_supplementary_data.docx]

***Online Supplemental Material***

**A novel machine learning-derived radiotranscriptomic signature of perivascular fat improves cardiac risk prediction using coronary CT angiography**

Evangelos K. Oikonomou^1,2^, Michelle C. Williams^3,4^, Christos P. Kotanidis^1,2^, Milind Y. Desai^5^, Mohamed Marwan^6^, Alexios S. Antonopoulos^1, 2^, Katharine E. Thomas^1, 2^, Sheena Thomas^1, 2^, Ioannis Akoumianakis^1^, Lampson M. Fan^7^, Sujatha Kesavan^7^, Laura Herdman^1, 2^, Alaa Alashi^5^, Erika Hutt Centeno^5^, Maria Lyasheva^1,2^, Brian P. Griffin^5^, Scott D. Flamm^5^, Cheerag Shirodaria^7,8^, Nikant Sabharwal^7^, Andrew Kelion^7^, Marc R. Dweck^3,4^, Edwin J.R. Van Beek^4^, John Deanfield^9^, Jemma C. Hopewell^10^_,_ Stefan Neubauer^1,11,12^, Keith M. Channon MD^1,11,12^, Stephan Achenbach MD^6^, David E. Newby^3,4^, Charalambos Antoniades^1, 2, 11,12*^

^1^ Division of Cardiovascular Medicine, Radcliffe Department of Medicine, University of Oxford, John Radcliffe Hospital, Headley Way, Oxford OX3 9DU, UK

^2.^ Oxford Academic Cardiovascular CT Core Laboratory, West Wing, John Radcliffe Hospital, Headley Way, Oxford OX3 9DU, UK

^3^ British Heart Foundation Centre for Cardiovascular Science, University of Edinburgh, Chancellor’s Building, 49 Little France Cres, Edinburgh EH16 4TJ, UK

^4^ Edinburgh Imaging Facility QMRI, University of Edinburgh, 47 Little France Cres, Edinburgh EH16 4TJ, UK

^5^ Heart and Vascular Institute, Cleveland Clinic, 9500 Euclid Avenue, Cleveland, OH 44195, USA

^6^ Department of Cardiology, Friedrich-Alexander-Universität Erlangen-Nürnberg, Ulmenweg 18, 91054, Erlangen, Germany

^7^ Department of Cardiology, Oxford University Hospitals NHS Foundation Trust, John Radcliffe Hospital, Oxford OX3 9DU, UK

^8^ Caristo Diagnostics Ltd, Whichford House, Parkway Court, John Smith Dr, Oxford OX4 2JY, UK

^9^ National Centre for Cardiovascular Prevention and Outcomes, Institute of Cardiovascular Science, University College London, 1 St Martins Le Grand, London EC1A 4NP, UK

^10^ Clinical Trial Service Unit, Nuffield Department of Population Health, University of Oxford, BHF Centre for Research Excellence, Big Data Institute, Old Road Campus, Roosevelt Drive, Oxford OX3 7LF, UK

^11^ British Heart Foundation Centre of Research Excellence, University of Oxford, John Radcliffe Hospital, Headley Way, Oxford OX3 9DU, UK

^12^ National Institute of Health Research Oxford Biomedical Research Centre, John Radcliffe Hospital, Headley Way, Oxford OX3 9DU, UK

**Supplemental Figures:** 5

**Supplemental Tables:** 12

***Corresponding author:**

Prof. Charalambos Antoniades MD PhD FESC

Professor of Cardiovascular Medicine

Division of Cardiovascular Medicine, Radcliffe Department of Medicine, University of Oxford

John Radcliffe Hospital, Oxford OX3 9DU, United Kingdom

Tel: +44-1865-221870

Fax: +44-1865-740352

e-mail: antoniad@well.ox.ac.uk

**Online Methods**

**1. Study Population Demographics**

Relevant demographic and clinical information for all patients included in Studies 1-4 of this study are presented in the following Tables S1-S4.

| **Table S1. Clinical characteristics of the Study 1 population.** | | | | |
| --- | --- | --- | --- | --- |
|  | | | **N (%) or median [25^th^-75^th^ percentile]** |  |
| **Total number** | | | **167** |  |
| Age (years) | | | 67 [59-74] |  |
| Body Mass Index (kg/m^2^) | | | 27.9 [25.2-31.1] |  |
| Female sex (n, %) | | | 27 (16.2) |  |
| Hypertension (n, %) | | | 123 (73.7) |  |
| Hypercholesterolaemia (n, %) | | | 147 (88.0) |  |
| Diabetes mellitus (n, %) | | | 40 (24.0) |  |
| Smoking (n, %) | |  | 11 (6.6) |  |
| Systolic BP (mmHg) | |  | 133 [118-144] |  |
| Diastolic BP (mmHg) | |  | 70 [63-80] |  |
| Total cholesterol (mg/dL) | |  | 126 [107-148] |  |
| High-density lipoprotein (HDL, mg/dL) | |  | 32 [27-38] |  |
| **Medications** | |  |  |  |
| Beta-blocker (n, %) | |  | 128 (76.6) |  |
| Statin (n, %) | | | 147 (88.0) |  |
| Antiplatelet (n, %) | | | 155 (92.8) |  |
| Calcium Channel Blocker (n, %) | | | 48 (28.7) |  |
| ACEi or ARB (n, %) | | | 110 (65.9) |  |
| **CAD presence (n, %)** | | | 167 (100) |  |
| **Tube voltage (n, %)** | *120 kVp* | | 167 (100) |  |
| ACEi: angiotensin-converting enzyme inhibitor; ARB: angiotensin-II-receptor blocker; BP: blood pressure; CAD: coronary artery disease; P values derived from Pearson’s Chi-square test for categorical variables and Mann-Whitney for continuous variables. | | | |  |

| **Table S2. Clinical characteristics of the Study 2 population.** | | | |
| --- | --- | --- | --- |
|  | **5-year MACE group** | **No-MACE group** | **P value** |
| **Total number** | **101** | **101** |  |
| Age (years) | 64 [55, 72] | 62 [53, 70] | 0.531 |
| Body Mass Index (kg/m^2^)* | 27.3 [25.1-32.2] | 27.7 [24.5-32.3] | 0.932 |
| Female sex | 34 (33.7) | 34 (33.7) | 1.000 |
| Hypertension (n, %) | 67 (66.3) | 62 (61.4) | 0.485 |
| Hypercholesterolaemia (n, %) | 40 (39.6) | 40 (39.6) | 0.936 |
| Diabetes mellitus (n, %) | 19 (18.8) | 15 (14.9) | 0.550 |
| Smoking (n, %) | 31 (30.7) | 25 (24.8) | 0.448 |
| Systolic BP (mmHg)* | 132 [120-141] | 136 [120-145] | 0.297 |
| Diastolic BP (mmHg)* | 76 [69-82] | 78.5 [70-84] | 0.374 |
| Total Cholesterol (mg/dL)* | 172 [140-216] | 205 [169-236] | 0.008 |
| High-density lipoprotein (mg/dL)* | 45 [37-53] | 46 [37-61] | 0.265 |
| Epicardial adipose tissue volume (cm^3^) | 96 [68-125] | 94 [69-122] | 0.420 |
| **Medications** |  |  |  |
| Beta-blocker (n, %) | 38 (37.6) | 36 (35.6) | 0.291 |
| Statin (n, %) | 52 (51.5) | 40 (39.6) | 0.197 |
| Antiplatelets (n, %) | 62 (61.4) | 54 (53.5) | 0.165 |
| Calcium Channel Blocker (n, %) | 22 (21.8) | 20 (19.8) | 0.705 |
| ACEi or ARB (n, %) | 37 (36.6) | 37 (36.6) | 0.766 |
| **CAD presence and severity (n, %)** |  |  | 0.011 |
| *No* | 14 (13.9) | 31 (30.7) |  |
| *Mild non-obstructive (<50%)* | 38 (37.6) | 40 (39.6) |  |
| *Moderate non-obstructive (50-69%)* | 23 (22.8) | 13 (12.9) |  |
| *Obstructive (≥70%)* | 26 (25.7) | 17 (16.8) |  |
| **Tube voltage (n, %)** |  |  | 1.000 |
| *120 kVp* | 69 (68.3) | 69 (68.3) |  |
| *100 kVp* | 32 (31.7) | 32 (31.7) |  |
| **Five-year clinical events** |  |  |  |
| Cardiac mortality (n, %) | 61 (60.4) | - | - |
| Non-fatal acute myocardial infarction (n, %) | 40 (39.6) | - | - |
| ACEi: angiotensin-converting enzyme inhibitor; ARB: angiotensin-II-receptor blocker; BP: blood pressure; CAD: coronary artery disease; FAI: fat attenuation index; HU: Hounsfield Units; LCA: left coronary artery; MACE: major adverse cardiac event; RCA: right coronary artery. P values derived from Pearson’s Chi-square test for categorical variables and Mann-Whitney for continuous variables. *Degree of missingness: 28.7% for Total Cholesterol, 39.1% for HDL, 21.8% for SBP/DBP, and 12.9% for BMI. Categorical variables are presented as numbers (percentages), while continuous variables are summarized as median [25th-75th percentile]. | | | |

| **Table S3. Clinical characteristics of the prospective SCOT-HEART population.** | |
| --- | --- |
|  | **N (%) or median [25^th^-75^th^ percentile]** |
| **Total number** | **1575** |
| Age (years) | 58 [51-65] |
| Body Mass Index (kg/m^2^) | 28.7 [25.7-32.4] |
| Female sex (n, %) | 687 (43.6) |
| Hypertension (n, %) | 539 (34.2) |
| Diabetes mellitus (n, %) | 176 (11.2) |
| Smoking (n, %) | 302 (19.8) |
| Systolic Blood Pressure (SBP, mmHg)* | 140 [126-152] |
| Diastolic Blood Pressure (DBP, mmHg)* | 80 [76-90] |
| Total cholesterol (mg/dL)* | 208 [174-239] |
| High-density lipoprotein (HDL, mg/dL)* | 50 [42-59] |
| **CAD presence and severity** |  |
| *No* | 571 (36.3) |
| *Mild non-obstructive (<50%)* | 336 (21.3) |
| *Moderate non-obstructive (50-69%)* | 275 (17.5) |
| *Obstructive (≥70%)* | 393 (25.0) |
| **High-risk plaque (HRP) feature presence** |  |
| *Any* | 610 (38.7) |
| *Spotty calcification* | 264 (16.8) |
| *Low-attenuation plaque* | 148 (9.4) |
| *Positive remodelling* | 527 (33.5) |
| *Napkin-ring sign* | 70 (4.4) |
| **Medications** |  |
| Beta-blocker (n, %) | 580 (36.3) |
| Statin (n, %) | 701 (44.5) |
| Antiplatelet (n, %) | 870 (55.2) |
| Calcium Channel Blocker (n, %) | 150 (9.5) |
| ACEi or ARB (n, %) | 256 (16.3) |
| **Tube voltage (n, %)** |  |
| *100 kVp* | 758 (48.1) |
| *120 kVp* | 817 (51.9) |
| **Follow-up and clinical events** |  |
| Follow-up duration (years) | 4.82 [4.18-5.72] |
| Deaths, n | 33 |
| Confirmed cardiac deaths, n | 1 |
| Non-fatal Acute Myocardial Infarction events, n | 33 |
| Cardiac death or non-fatal AMI (MACE), n | 34 |
| Late revascularization (≥6 weeks)**, n | 176 |
| MACE or late revascularization, n | 188 |
| ACEi: angiotensin-converting enzyme inhibitor; AMI: acute myocardial infarction; ARB: angiotensin-II-receptor blocker; CAD: coronary artery disease; FAI: fat attenuation index; HDL: high-density lipoprotein; HU: Hounsfield Units; LCA: left coronary artery; MACE: major adverse cardiac event; RCA: right coronary artery; SCOT-HEART: Scottish COmputed Tomography of the HEART trial. P values derived from Pearson’s Chi-square test for categorical variables and Mann-Whitney for continuous variables. *Degree of missingness: Systolic blood pressure = 0.8%, Total cholesterol = 7.4%, HDL =28.1%. **All revascularization events were percutaneous coronary intervention. | |

| **Table S4. Clinical characteristics of Study 3.** | | | | |
| --- | --- | --- | --- | --- |
|  |  | **Stable CAD** | **AMI** | **P value for matching** |
| **Total number** |  | **44** | **44** | **-** |
| Age (years) |  | 62 [51-70] | 62 [53-72] | 0.363 |
| BMI (kg/m^2^) |  | 27.8 [24.3-31.0] | 26.8 [24.1-31.9] | 0.867 |
| Male sex (n, %) |  | 28 (63.6) | 37 (84.1) | 0.052 |
| Hypertension (n, %) |  | 17 (38.6) | 25 (56.8) | 0.135 |
| Hypercholesterolaemia (n, %) |  | 16 (36.4) | 25 (56.8) | 0.087 |
| Diabetes mellitus (n, %) |  | 5 (11.4) | 8 (18.2) | 0.548 |
| Smoking |  |  |  | 0.300 |
| *Active* (n, %) |  | 2 (4.5) | 3 (6.8) |  |
| *Past* (n, %) |  | 22 (50.0) | 28 (63.6) |  |
| **Medications** |  |  |  |  |
| Beta-blocker (n, %) |  | 10 (22.7) | 8 (18.2) | 0.792 |
| Statin (n, %) |  | 15 (34.1) | 15 (34.1) | 1.000 |
| Antiplatelet (n, %) |  | 11 (25.0) | 10 (22.7) | 1.000 |
| CCB (n, %) |  | 5 (11.4) | 8 (18.2) | 0.548 |
| ACEi or ARB (n, %) |  | 15 (34.1) | 12 (27.3) | 0.644 |
| **Tube voltage** (n, %) |  |  |  | 0.145 |
| *120 kVp* |  | 34 (77.3) | 40 (90.9) |  |
| *100 kVp* |  | 10 (22.7) | 4 (9.1) |  |
| **FRP (AU)** |  | 0.45 [0.42-0.49] | 0.55 [0.49-0.63] | <0.001 |
| ACEi: angiotensin-converting enzyme inhibitor; ARB: angiotensin-II-receptor blocker; AU: arbitrary units; BMI: body mass index; CCB: calcium-channel blocker; FRP: Fat Radiomic Profile. P values derived from Pearson’s Chi-square test for categorical variables and Mann-Whitney for continuous variables. Categorical variables are presented as numbers (percentages), while continuous variables are summarized as median [25^th^-75^th^ percentile]. | | | | |

**2. Coronary computed tomography angiography (CCTA) acquisition protocol**

**Study 1:** Participants in Study 1 underwent CCTA using a 64-slice scanner (LightSpeed Ultra, General Electric), as previously described.^1^ Heart rate was optimised using intravenous injection of beta-blockers and sublingual glyceryl-trinitrate (800ug) was also administered to achieve maximum coronary vasodilatation. A non-contrast prospectively ECG triggered axial acquisition CT scan was obtained (0.35 sec rotation time, 2.5 mm axial slice thickness, 20 mm detector coverage, tube energy of 120 kV and 200 mA) with the carina and the diaphragm used as cranial and caudal landmarks respectively. Lung field of view was extended to cover the entire thoracic soft tissue (for subcutaneous adipose tissue analysis). CCTA was performed following intravenous injection of 95ml of iodine based contrast medium (Niopam 370, BRACCO) at a flow rate rate of 6mL/sec (tube energy of 120 kVp, axial slice thickness of 0.625 mm, rotation time of 0.35 sec, detector coverage of 40 mm). Prospective image acquisition was used by ECG-gating at 75% of cardiac cycle (with 100 msec padding for optimal imaging of the right coronary artery if required).

**Study 2:**

**Cleveland arm (CRISP-CT study):** CCTA scans were performed using either a 256-slice Brilliance iCT scanner (Philips Medical Systems, Best, The Netherlands), a 2 x 128-slice Definition Flash scanner (Siemens Healthcare, Erlangen, Germany) or a 2 x 192-slice Somatom Force CT scanner (Siemens Healthcare, Forchheim, Germany). In patients with heart rate > 60 beats/minute, 5 mg of intravenous metoprolol (with incremental 5 mg doses up to a maximum dose of 30 mg) or intravenous diltiazem (5 mg increments up to 20 mg maximum), if the heart rate remained above 60 beats per minute once the patient was positioned on the CT table. Patients also received 0.3 mg of nitroglycerin sublingually immediately before CCTA and iodinated contrast (Omnipaque 350, General Electric, Milwaukee, USA) was administered at flow rate of 5-6 ml/s.^2^

**Erlangen arm (CRISP-CT study):** CCTA scans were performed using either a 2 x 64-slice scanner (Definition Flash, Siemens Healthcare, Forchheim, Germany), a 64-slice (Siemens Sensation 64, Siemens Healthcare, Forchheim, Germany) or a 2 x 128-slice scanner (Somatom Definition Flash, Siemens Healthcare, Forchheim, Germany). Oral medication with 100 mg atenolol was administered one hour before CT if heart rate was > 60 beats per minute with additional 5 mg doses of metoprolol intravenously up to a maximum dose of 30 mg, if the heart rate remained above 60 beats per minute once the patient was positioned on the CT table. Patients also received 0.8 mg of nitroglycerine sublingually immediately before CCTA and iodinated contrast (Omnipaque 350, Schering AG, Berlin, Germany) was administered at flow rate of 5-6 ml/s.^2^

**SCOT-HEART study:** CCTA scans were performed using either two 64 detector row scanners (Brilliance 64, Philips Medical Systems, Netherlands, and Biograph mCT, Siemens, Germany) and one 320 detector row scanner (Aquilion ONE, Toshiba Medical Systems, Japan) at three imaging sites according to each site’s local protocol.^3, 4^ A total of 1786 patients were available for analysis. In accordance with our previous studies, scans performed at tube voltage settings other than 100 or 120 kVp, and scans with missing slices, beam hardening or step artifacts were excluded from the analysis (n=211), resulting in a study dataset of 1575 individual CCTA scans.

**Study 3:** Participants in Study 3 underwent CCTA using a 64-slice scanner (General Electric, LightSpeed Ultra, General Electric, Milwaukee, WI, USA). Heart rate was optimised using intravenous injection of beta-blockers and sublingual glyceryl-trinitrate (800ug) was also administered to achieve maximum coronary vasodilatation. CCTA was performed following intravenous injection of 50-70 ml of iodine based contrast medium (Niopam 370, BRACCO UK Ltd) at a flow rate rate of 5.5-6.5 mL/sec (axial slice thickness of 0.625 mm, rotation time of 0.35 sec, detector coverage of 40 mm, tube energy selected based on body habitus and according to local clinical practice). Prospective image acquisition was used by ECG-gating at 75% of cardiac cycle (with 100msec padding if required). If not possible, retrospective image acquisition was used (e.g. in the presence of irregular rhythm).

**3. Computed tomography image analysis and radiomic phenotyping**

***3i) Adipose tissue radiomic characterization***

***Radiomic characterization***

In each CCTA scan (Studies 2-3) the two segmented PVAT volumes were used to calculate 843 radiomic features (1686 features in total) (**Table S5**), using the SlicerRadiomics extension which incorporates the Pyradiomics library into 3D Slicer.^5^ Shape-related and first-order radiomic features were calculated using the raw HU values of the segmented PVAT. For calculation of texture features (Gray Level Co-occurrence Matrix [GLCM], Gray Level Dependence Matrix [GLDM], Gray Level Run-Length Matrix [GLRLM], Gray Level Size Zone Matrix [GLSZM], and Neighbouring Gray Tone Difference Matrix [NGTDM], **Tables S6-12**), AT voxels were discretized into 16 bins of equal width (width of ten HU), to reduce noise while allowing a sufficient resolution to detect biologically significant spatial changes in PVAT attenuation. Discretization into 16 bins has been previously recommended as the optimal approach to increase the signal-to-noise ratio of images for radiomic analysis.^6, 7^ To enforce symmetrical, rotationally-invariant results, texture statistics (GLCM etc.) were calculated in all directions and then averaged, as previously described.^7^

First order and texture-based statistics were also calculated for three-dimensional wavelet transformations of the original image resulting in eight additional sets of radiomic features.^8^ Wavelet transformation decomposes the data into high and low-frequency components. At high frequency (shorter time intervals), the wavelets can capture discontinuities, ruptures and singularities in the original data. At low frequency (longer time intervals), the wavelet characterizes the coarse structure of the data to identify the long-term trends. Thus, the wavelet analysis allows extraction of hidden and significant temporal features of the original data, while improving the signal-to-noise ratio of imaging studies.^8, 9^

***Epicardial adipose tissue volume***

The total epicardial adipose tissue (EAT) volume was assessed in a semi-automated manner by tracking the contour of the pericardium from the level of the pulmonary artery bifurcation to the apex of the heart at the most caudal end.^1, 2^

***Perivascular Fat Attenuation Index (FAI)***

Perivascular FAI (measured around the proximal 10-50 mm of the right coronary artery or around a given coronary lesion, as validated in our previous work)^1, 2^ were also quantified in the OXACCT core lab using dedicated software (Aquarius Workstation® V.4.4.11-13, TeraRecon Inc., Foster City, CA, USA) for basic segmentation and CaRi-HEART proprietary algorithms (Caristo Diagnostics, Oxford UK) for final calculation.^2^

***3ii) Coronary artery disease (CAD)***

Coronary artery disease was defined as luminal stenosis more than 50% in one or more major epicardial vessels.

***3iii) Agatston coronary calcium scoring (CCS)***

Coronary calcium scoring was performed in non-contrast-enhanced scans using dedicated software (VScore, Vital Images, Minnetonka, USA or scanner console software). Agatston score was calculated using a threshold of 130 HU (Hounsfield units) for each vessel and summed to give a total score.^10^

***3iv) High-risk plaque (HRP) features***

High-risk plaque (HRP) features were defined as the presence of at least one of the following: low-attenuation plaque, positive remodelling, napkin-ring sign or spotty calcification. The definitions for each one of these adverse plaque characteristics have been previously described.^11, 12^

***3v) Mean arterial lumen attenuation***

The mean arterial lumen attenuation was measured on an axial slice by placing a circular region of interest (ROI) in the lumen of the aortic root at the level of the origin of the left main coronary artery.

**4. Case-control matching**

**Study 2:** In the discovery CCTA sample, consisting of scans collected from the CRISP-CT and SCOT-HEART studies, one-to-one matching was performed for the cohorts, including age, gender, CT scanner used and tube voltage, whereas a nearest matching approach was used for traditional cardiovascular risk factors (hypertension, hypercholesterolemia, diabetes mellitus, smoking), body mass index (BMI), epicardial adipose tissue volume and baseline medications.

**Study 3:** Using a pool of 93 patients with suspected stable CAD that underwent CCTA as part of the Oxford Risk Factors and Non-invasive imaging (ORFAN) study, a nearest-match method was applied to identify a subgroup of 44 patients with matched demographics to the 44 acute myocardial infarction patients included in this Study. Matching was performed for age, gender, hypertension, hypercholesterolemia, diabetes mellitus, smoking, body mass index (BMI), epicardial adipose tissue volume and baseline medications.

**5. Gene expression studies (Study 1)**

***Tissue collection, RNA (ribonucleic acid) isolation and reverse transcription***

Subcutaneous adipose tissue specimens were collected during surgery from the incision site and stored in TRI reagent (Sigma, catalogue number T9424) at -80oC until thawed for RNA isolation.

This depot was selected for radiotranscriptomic analysis, given that its radiomic attenuation and texture is influenced by biological signals generated within the tissue (captured by the expression of genes within the tissue itself), unlike depots such as epicardial or perivascular fat where inflammatory signals may originate from the adjacent vessels in a paracrine manner, and can not be captured by gene expression studies using fat biopsies.^1, 13, 14^

Total RNA was isolated by a phenol to chloroform (1:5 ratio) separation protocol followed by a magnetic beads-based RNA purification method on a KingFischer magnetic particle processor (Thermo Fischer Scientific), using the MagMAX mirVana total RNA isolation kit (Thermo Fischer Scientific, Catalogue Number A27828). RNA concentration and integrity were assessed spectrophotometrically on NanoDrop ND-1000. For reverse transcription of RNA to cDNA (complementary deoxyribonucleic acid) we used the SuperScript VILO mastermix (Thermo Fischer Scientific) adhering to manufacturer’s instructions and extending the cDNA synthesis step to two hours at 60oC on a Veriti thermal cycler (ABI).

***Quantitative real-time PCR***

Quantitative real-time polymerase chain reaction (PCR) was performed on a QuantStudio 7 flex real-time PCR system (Thermo Fischer Scientific) perusing TaqMan chemistry, in accordance with the standard universal TaqMan protocol, as indicated by the manufacturer. Using a starting mass of 5ng of cDNA, all samples were run in duplicates, and data was analysed by the Pfaffl method (you can use this as reference here). Cyclophilin A (*PPIA*) was used as the housekeeping gene for all human adipose tissue samples. TaqMan probes IDs are as follows: *PPIA*: Hs99999904_m1; *PECAM1* (*CD31*): Hs01065279_m1; *TNF*: Hs01065279_m1; *COL1A1*: Hs00164004_m1.

**6. Statistical analysis (supplementary information)**

***Study 2 (random forest model):*** To address the multi-dimensionality and possible redundancy of the radiomic dataset, the amount of pairwise correlations was reduced at the level of |rho|≥0.9 using the *findCorrelation* function of the *caret* package in R. In short, the absolute values of all pairwise correlations were considered. If two variables were highly correlated (|rho|≥0.9), the function looked at the mean absolute correlation of each variable and removed the variable with the largest mean absolute correlation. In an initial assessment of the dataset, a Manhattan plot was created based on the value of each radiomic feature in discriminating the MACE from the non-MACE cases (graphical representation of the negative logarithm [log_10_] of the P value from univariable receiver operating characteristic curve [ROC] analysis). Subsequently, the nested case-control study in Study 2 was randomly split in a training & internal validation set (80%) and an external validation set (20%), ensuring an equal representation of events and non-events in both splits. A random forest method was selected a priori to train a model to discriminate 5-year MACE from no MACE, since it enables modelling of non-linear relationships, can train on small datasets, is less sensitive to outliers and has been previously used in patient-specific predictive modelling.^15^ All radiomic features were centered and scaled prior to inclusion in the models. The optimal number of features in the model was estimated using a recursive feature elimination approach (with 4, 8, 16, 32, 64, 128, 256 and maximum number of features) and based on the model’s accuracy on repeated five-fold cross validation (three repeats). Following internal validation, the model was further validated by measuring the C-statistic in the remaining (validation) dataset. Missingness for factors included in the models was addressed by creating a third group for categorical variables (yes/no/unknown) and median imputation for continuous variables (i.e. total cholesterol and high-density lipoprotein levels).

***Study 3 (FAI-associated plaque inflammatory burden):*** FAI-associated plaque inflammatory burden was determined by the area under the curve of perivascular FAI measured along a defined lesion (including the proximal, mid and distal stented tertiles and 5mm-long proximal and distal reference segments). This is equal to the polygonal area defined by the aforementioned curve and a horizontal line crossing the y axis at the level of -190 HU, the lowest attenuation value attributed to adipose tissue on CT.

**7. Perivascular Adipose Tissue (PVAT) radiomic feature definition**

**(Adapted from** [**http://pyradiomics.readthedocs.io/en/latest/features.html**](http://pyradiomics.readthedocs.io/en/latest/features.html) **– last accessed March 22, 2018)**

**The definitions of the radiomic features have been derived from the pyradiomics library and the pyradiomics community maintains the copyright for the definitions mentioned below (© Copyright 2016, pyradiomics community,** [**http://github.com/radiomics/pyradiomics**](http://github.com/radiomics/pyradiomics) **Revision eae15eff, last accessed July 27, 2019).**

**The pyradiomics package is covered by the open source 3-clause BSD License (© 2017 Harvard Medical School:** [**https://github.com/Radiomics/pyradiomics/blob/master/LICENSE.txt**](https://github.com/Radiomics/pyradiomics/blob/master/LICENSE.txt)**, last accessed on July 27, 2019)**

In our analysis, we measured a total of 843 radiomic features in PVAT, as summarized in Table S5.

| **Table S5. Breakdown of PVAT radiomic features.** | | | |
| --- | --- | --- | --- |
|  | **Original** | **Wavelets transformations (n=8)** | **All** |
| **First order** | 18 | 144 | 162 |
| **Shape-related** | 15 | - | 15 |
| **GLCM** | 23 | 184 | 207 |
| **GLDM** | 14 | 112 | 126 |
| **GLRLM** | 16 | 128 | 144 |
| **GLSZM** | 16 | 128 | 144 |
| **NGTDM** | 5 | 40 | 45 |
| **Total** | 107 | 736 | 843 |
| GLCM: gray level co-occurrence matrix; GLDM: gray level dependence matrix; GLRLM: gray level run length matrix; GLSZM: gray level size zone matrix; NGTDM: neighbouring gray tone dependence matrix; PVAT: perivascular adipose tissue. | | | |

***a. First Order Statistics***

These statistics describe the central tendency, variability, uniformity, asymmetry, skewness and magnitude of the attenuation values in a given region of interest (ROI), disregarding the spatial relationship of the individual voxels. As such, they describe quantitative and qualitative features of the whole ROI (PVAT). A total of 19 features were calculated for each one of the eight wavelet transformations and the original CT image (Table S6), as follows:

**Let:**

- ***X*** be a set of ***N_p_*** voxels included in the region of interest (ROI)
- ***P(i)*** be the first order histogram with ***N_g_***discrete intensity levels, where ***N_g_*** is the number of non-zero bins, equally spaced from 0 with a width.
- ***p(i)*** be the normalized first order histogram and equal to $\frac{\text{P}\boldsymbol{(i)}}{\boldsymbol{N}_{\boldsymbol{p}}}$
- **c** is a value that shifts the intensities to prevent negative values in X. This ensures that voxels with the lowest gray values contribute the least to Energy, instead of voxels with gray level intensity closest to 0. Since the HU range of AT (-190 to -30 HU) does not include zero, c was set at c=0. Therefore, higher energy corresponds to less radiodense AT, and therefore a higher lipophilic content.
- ***ϵ*** is an arbitrarily small positive number (≈2.2×10^−16^)

| **Table S6. First-order radiomic features for PVAT characterization** | |
| --- | --- |
| **Radiomic feature** | **Interpretation** |
| $\mathbf{E}\text{nergy}=\sum_{i=1}^{N_{p}} (\text{X}(i)+c)^{2}$ | **Energy** is a measure of the magnitude of voxel values in an image. A larger value implies a greater sum of the squares of these values. |
| $\text{Total Energy}=V_{voxel}\sum_{i=1}^{N_{p}} (\text{X}(i)+c)^{2}$ | **Total Energy** is the value of Energy feature scaled by the volume of the voxel in cubic mm. |
| $\text{Entropy}=-\sum_{i=1}^{N_{g}} p(i)\log_{2}(p(i)+\epsilon)$ | **Entropy** specifies the uncertainty/randomness in the image values. It measures the average amount of information required to encode the image values |
| $\text{Minimum}=min(\text{X})$ | The **minimum** gray level intensity within the ROI. |
| The **10th percentile** of X | The **10th percentile** of X |
| The **90th percentile** of X | The **90th percentile** of X |
| $\text{Maximum}=max(\text{X})$ | The **maximum** gray level intensity within the ROI. |
| $\text{Mean}=\frac{1}{N_{p}}\sum_{i=1}^{N_{p}} \text{X}(i)$ | The average (**mean**) gray level intensity within the ROI. |
| **Median** | The **median** gray level intensity within the ROI. |
| $\text{Interquartile range}=\text{P}_{75}-\text{P}_{25}$ | Here P_25_ and P_75_ are the 25^th^ and 75^th^ percentile of the image array, respectively. |
| $\text{Range}=max(\text{X})-min(\text{X})$ | The range of gray values in the ROI. |
| $\text{MAD}=\frac{1}{N_{p}}\sum_{i=1}^{N_{p}} \vert\text{X}(i)-\overline{X}\vert$ | **Mean Absolute Deviation (MAD)** is the mean distance of all intensity values from the Mean Value of the image array. |
| $\text{rMAD}=\frac{1}{N_{10-90}}\sum_{i=1}^{N_{10-90}} \vert\text{X}_{10-90}(i)-\overline{X}_{10-90}\vert$ | **Robust Mean Absolute Deviation (rMAD)** is the mean distance of all intensity values from the Mean Value calculated on the subset of image array with gray levels in between, or equal to the 10^th^ and 90^th^ percentile. |
| $\text{RMS}=\sqrt{\frac{1}{N_{p}}\sum_{i=1}^{N_{p}} (\text{X}(i)+c)^{2}}$ | **Root Mean Squared (RMS)** is the square-root of the mean of all the squared intensity values. It is another measure of the magnitude of the image values. This feature is volume-confounded, a larger value of c increases the effect of volume-confounding. |
| $\text{Skewness}=\frac{\mu_{3}}{\sigma^{3}}=\frac{\frac{1}{N_{p}}\sum_{i=1}^{N_{p}} (\text{X}(i)-\overline{X})^{3}}{{(\sqrt{\frac{1}{N_{p}}\sum_{i=1}^{N_{p}} (\text{X}(i)-\overline{X})^{2}})}^{3}}$ | **Skewness** measures the *asymmetry* of the distribution of values about the Mean value. Depending on where the tail is elongated and the mass of the distribution is concentrated, this value can be positive or negative. (Where μ^3^ is the 3^rd^ central moment). |
| $\text{Kurtosis}=\frac{\mu_{4}}{\sigma^{4}}=\frac{\frac{1}{N_{p}}\sum_{i=1}^{N_{p}} (\text{X}(i)-\overline{X})^{4}}{{(\frac{1}{N_{p}}\sum_{i=1}^{N_{p}} (\text{X}(i)-\overline{X})^{2})}^{2}}$ | **Kurtosis** is a measure of the ‘*peakedness*’ of the distribution of values in the image ROI. A higher kurtosis implies that the mass of the distribution is concentrated towards the tail(s) rather than towards the mean. A lower kurtosis implies the reverse: that the mass of the distribution is concentrated towards a spike near the Mean value. (Where μ4 is the 4th central moment). |
| $\text{Variance}=\frac{1}{N_{p}}\sum_{i=1}^{N_{p}} (\text{X}(i)-\overline{X})^{2}$ | **Variance** is the the mean of the squared distances of each intensity value from the Mean value. This is a measure of the spread of the distribution about the mean. |
| $\text{Uniformity}=\sum_{i=1}^{N_{g}} p(i)^{2}$ | **Uniformity** is a measure of the sum of the squares of each intensity value. This is a measure of the heterogeneity of the image array, ***where a greater uniformity implies a greater heterogeneity or a greater range of discrete intensity values****.* |
| Adapted from <http://pyradiomics.readthedocs.io/en/latest/features.html> – last accessed March 22, 2018. © Copyright 2016, pyradiomics community, http://github.com/radiomics/pyradiomics Revision eae15eff. | |

***b. Shape-related Statistics***

Shape-related statistics describe the size and shape of a given ROI, without taking into account the attenuation values of its voxels. Since they are independent of the gray level intensities, shape-related statistics were consistent across all wavelet transformation and the original CT image, and therefore were only calculated once. These were defined as follows (Table S7):

Let:

**V** the volume of the ROI in mm^3^

**A** the surface area of the ROI in mm^2^

| **Table S7. Shape-related radiomic features for PVAT characterization** | |
| --- | --- |
| **Radiomic feature** | **Interpretation** |
| $\mathbf{Volume}=\sum_{i=1}^{N} V_{i}$ | The **volume** of the ROI V is approximated by multiplying the number of voxels in the ROI by the volume of a single voxel **V_i_**. |
| $\mathbf{Surface Area}=\sum_{i=1}^{N} \frac{1}{2}\vert\text{a}_{i}\text{b}_{i}\times\text{a}_{i}\text{c}_{i}\vert$ | **Surface Area** is an approximation of the surface of the ROI in mm^2^, calculated using a marching cubes algorithm, where ***N*** is the number of triangles forming the surface mesh of the volume (ROI), ***a_i_b_i_*** and ***a_i_c_i_*** are the edges of the ***i^th^*** triangle formed by points ***a_i_, b_i_*** and ***c_i._*** |
| $\text{Surface to volume ratio}=\frac{A}{V}$ | Here, a lower value indicates a more compact (sphere-like) shape. This feature is not dimensionless, and is therefore (partly) dependent on the volume of the ROI. |
| $\text{Sphericity}=\frac{\sqrt[3]{36\pi V^{2}}}{A}$ | **Sphericity** is a measure of the roundness of the shape of the tumor region relative to a sphere. It is a dimensionless measure, independent of scale and orientation. The value range is 0<sphericity≤1, where a value of 1 indicates a perfect sphere (a sphere has the smallest possible surface area for a given volume, compared to other solids). |
| **Volume Number** | Total number of discrete volumes in the ROI. |
| **Voxel Number** | Total number of discrete voxels in the ROI. |
| **Maximum 3D diameter** | Maximum 3D diameter is defined as the largest pairwise Euclidean distance between surface voxels in the ROI (Feret Diameter). |
| **Maximum 2D diameter (Slice)** | Maximum 2D diameter (Slice) is defined as the largest pairwise Euclidean distance between ROI surface voxels in the row-column (generally the axial) plane. |
| **Maximum 2D diameter (Column)** | Maximum 2D diameter (Column) is defined as the largest pairwise Euclidean distance between ROI surface voxels in the row-slice (usually the coronal) plane. |
| **Maximum 2D diameter (Row)** | Maximum 2D diameter (Row) is defined as the largest pairwise Euclidean distance between tumor surface voxels in the column-slice (usually the sagittal) plane. |
| $\text{Major axis}=4\sqrt{\lambda_{\text{major}}}$ |  |
| $\text{Minor axis}=4\sqrt{\lambda_{\text{minor}}}$ |  |
| $\text{Least axis}=4\sqrt{\lambda_{\text{least}}}$ |  |
| $\text{Elongation}=\sqrt{\frac{\lambda_{\text{minor}}}{\lambda_{\text{major}}}}$ | Here, **λ_major_** and **λ_minor_** are the lengths of the largest and second largest principal component axes. The values range between 1 (circle-like (non-elongated)) and 0 (single point or 1 dimensional line). |
| $\text{Flatness}=\sqrt{\frac{\lambda_{\text{least}}}{\lambda_{\text{major}}}}$ | Here, **λ_major_** and **λ_minor_** are the lengths of the largest and smallest principal component axes. The values range between 1 (non-flat, sphere-like) and 0 (a flat object). |
| Adapted from <http://pyradiomics.readthedocs.io/en/latest/features.html> – last accessed March 22, 2018. © Copyright 2016, pyradiomics community, http://github.com/radiomics/pyradiomics Revision eae15eff. | |

***c. Gray Level Co-occurrence Matrix (GLCM)***

In simple words, a GLCM describes the number of times a voxel of a given attenuation value ***i*** is located next to a voxel of ***j***. A GLCM of size ***N_g_***×***N_g_*** describes the second-order joint probability function of an image region constrained by the mask and is defined as P(***i,j***|***δ,θ***). The (***i,j***)^th^ element of this matrix represents the number of times the combination of levels ***i*** and ***j*** occur in two pixels in the image, that are separated by a distance of ***δ*** pixels along angle ***θ***. The distance ***δ*** from the center voxel is defined as the distance according to the infinity norm. For ***δ=1***, this results in 2 neighbors for each of 13 angles in 3D (26-connectivity) and for ***δ=2*** a 98-connectivity (49 unique angles). In order to get rotationally invariant results, statistics are calculated in all directions and then averaged, to ensure a symmetrical GLCM (Table S8).

Let:

**ϵ** be an arbitrarily small positive number **(≈2.2×10−16**)

**P(*i,j*)** be the co-occurence matrix for an arbitrary ***δ*** and ***θ***

**p(*i,j*)** be the normalized co-occurence matrix and equal to $\frac{\text{P}(i,j)}{\sum\text{P}(i,j)}$

Ng be the number of discrete intensity levels in the image

$\boldsymbol{p}_{\boldsymbol{x}}\boldsymbol{(i)=}\sum_{\boldsymbol{j=1}}^{\boldsymbol{N}_{\boldsymbol{g}}} \boldsymbol{P(i,j)}$ be the marginal row probabilities

$\boldsymbol{p}_{\boldsymbol{y}}\boldsymbol{(j)=}\sum_{\boldsymbol{i=1}}^{\boldsymbol{N}_{\boldsymbol{g}}} \boldsymbol{P(i,j)}$ be the marginal column probabilities

***μ_x_*** be the mean gray level intensity of ***p_x_*** and defined as $\boldsymbol{\mu}_{\boldsymbol{x}}\boldsymbol{=}\sum_{\boldsymbol{i=1}}^{\boldsymbol{N}_{\boldsymbol{g}}} \boldsymbol{p}_{\boldsymbol{x}}\boldsymbol{(i)i}$

***μ_y_*** be the mean gray level intensity of ***p_y_*** and defined as $\boldsymbol{\mu}_{\boldsymbol{y}}\boldsymbol{=}\sum_{\boldsymbol{j=1}}^{\boldsymbol{N}_{\boldsymbol{g}}} \boldsymbol{p}_{\boldsymbol{y}}\boldsymbol{(j)j}$

***σ_x_*** be the standard deviation of ***p_x_***

***σ_y_*** be the standard deviation of ***p_y_***

$$p_{x+y}(k)=\sum_{i=1}^{N_{g}} \sum_{j=1}^{N_{g}} p(i,j),\text{ where }i+j=k,\text{ and }k=2,3,\ldots,2N_{g}$$

$$p_{x-y}(k)=\sum_{i=1}^{N_{g}} \sum_{j=1}^{N_{g}} p(i,j),\text{ where }|i-j|=k,\text{ and }k=0,1,\ldots,N_{g}-1$$

$HX=-\sum_{i=1}^{N_{g}} p_{x}(i)\log_{2}(p_{x}(i)+\epsilon)$ be the entropy of ***p_x_***

$HY=-\sum_{j=1}^{N_{g}} p_{y}(j)\log_{2}(p_{y}(j)+\epsilon)$ be the entropy of ***p_y_***

$$HXY1=-\sum_{i=1}^{N_{g}} \sum_{j=1}^{N_{g}} p(i,j)\log_{2}(p_{x}(i)p_{y}(j)+\epsilon)$$

$$HXY2=-\sum_{i=1}^{N_{g}} \sum_{j=1}^{N_{g}} p_{x}(i)p_{y}(j)\log_{2}(p_{x}(i)p_{y}(j)+\epsilon)$$

For distance weighting, GLCM matrices are weighted by weighting factor W and then summed and normalised.. Weighting factor W is calculated for the distance between neighbouring voxels by $\boldsymbol{W=}\boldsymbol{e}^{\boldsymbol{-\|d}\boldsymbol{\|}^{\boldsymbol{2}}}$, where d is the distance for the associated angle.

| **Table S8. Gray Level Co-occurrence Matrix (GLCM) statistics for PVAT characterization** | |
| --- | --- |
| **Radiomic feature** | **Interpretation** |
| $\text{Autocorrelation}=\sum_{i=1}^{N_{g}} \sum_{j=1}^{N_{g}} p(i,j)ij$ | **Autocorrelation** is a measure of the magnitude of the fineness and coarseness of texture. |
| $\text{Joint average}=\mu_{x}=\sum_{i=1}^{N_{g}} \sum_{j=1}^{N_{g}} p(i,j)i$ | Returns the mean gray level intensity of the i distribution. |
| $\text{Cluster prominence}=\sum_{i=1}^{N_{g}} \sum_{j=1}^{N_{g}} (i+j-\mu_{x}-\mu_{y})^{4}p(i,j)$ | **Cluster Prominence** is a measure of the skewness and asymmetry of the GLCM. A higher value implies more asymmetry around the mean while a lower value indicates a peak near the mean value and less variation around the mean. |
| $\text{Cluster tendency}=\sum_{i=1}^{N_{g}} \sum_{j=1}^{N_{g}} (i+j-\mu_{x}-\mu_{y})^{2}p(i,j)$ | **Cluster Tendency** is a measure of groupings of voxels with similar gray-level values. |
| $\text{Cluster shade}=\sum_{i=1}^{N_{g}} \sum_{j=1}^{N_{g}} (i+j-\mu_{x}-\mu_{y})^{3}p(i,j)$ | **Cluster Shade** is a measure of the skewness and uniformity of the GLCM. A higher cluster shade implies greater asymmetry about the mean. |
| $\text{Contrast}=\sum_{i=1}^{N_{g}} \sum_{j=1}^{N_{g}} (i-j)^{2}p(i,j)$ | **Contrast** is a measure of the local intensity variation, favoring values away from the diagonal (i=j). A larger value correlates with a greater disparity in intensity values among neighboring voxels. |
| $\text{Correlation}=\frac{\sum_{i=1}^{N_{g}} \sum_{j=1}^{N_{g}} p(i,j)ij-\mu_{x}\mu_{y}}{\sigma_{x}(i)\sigma_{y}(j)}$ | **Correlation** is a value between 0 (uncorrelated) and 1 (perfectly correlated) showing the linear dependency of gray level values to their respective voxels in the GLCM. |
| $\text{Difference average}=\sum_{k=0}^{N_{g}-1} kp_{x-y}(k)$ | **Difference Average** measures the relationship between occurrences of pairs with similar intensity values and occurrences of pairs with differing intensity values. |
| $\text{Difference entropy}=\sum_{k=0}^{N_{g}-1} p_{x-y}(k)\log_{2}(p_{x-y}(k)+\epsilon)$ | **Difference Entropy** is a measure of the randomness/variability in neighborhood intensity value differences. |
| $\text{Difference variance}=\sum_{k=0}^{N_{g}-1} (k-DA)^{2}p_{x-y}(k)$ | **Difference Variance** is a measure of heterogeneity that places higher weights on differing intensity level pairs that deviate more from the mean. |
| $\text{Joint energy}=\sum_{i=1}^{N_{g}} \sum_{j=1}^{N_{g}} (p(i,j))^{2}$ | **Joint energy** is a measure of homogeneous patterns in the image. A greater **joint energy** implies that there are more instances of intensity value pairs in the image that neighbor each other at higher frequencies. (also known as Angular Second Moment). |
| $\text{Joint entropy}=-\sum_{i=1}^{N_{g}} \sum_{j=1}^{N_{g}} p(i,j)\log_{2}(p(i,j)+\epsilon)$ | **Joint entropy** is a measure of the randomness/variability in neighborhood intensity values. |
| $\text{IMC 1}=\frac{HXY-HXY1}{\max\left\{ HX,HY \right\}}$ | **Informational measure of correlation 1** |
| $\text{IMC 2}=\sqrt{1-e^{-2(HXY2-HXY)}}$ | **Informational measure of correlation 2** |
| $\text{IDM}=\sum_{i=1}^{N_{g}} \sum_{j=1}^{N_{g}} \frac{p(i,j)}{1+\vert i-j\vert^{2}}$ | IDM (**inverse difference moment** a.k.a Homogeneity 2) is a measure of the local homogeneity of an image. IDM weights are the inverse of the Contrast weights (decreasing exponentially from the diagonal i=j in the GLCM). |
| $\text{IDMN}=\sum_{i=1}^{N_{g}} \sum_{j=1}^{N_{g}} \frac{p(i,j)}{1+(\frac{\vert i-j\vert^{2}}{N_{g}^{2}})}$ | IDMN (**inverse difference moment normalized**) is a measure of the local homogeneity of an image. IDMN weights are the inverse of the Contrast weights (decreasing exponentially from the diagonal i=j in the GLCM). Unlike Homogeneity2, IDMN normalizes the square of the difference between neighboring intensity values by dividing over the square of the total number of discrete intensity values. |
| $\text{ID}=\sum_{i=1}^{N_{g}} \sum_{j=1}^{N_{g}} \frac{p(i,j)}{1+\vert i-j\vert}$ | ID (**inverse difference** a.k.a. Homogeneity 1) is another measure of the local homogeneity of an image. With more uniform gray levels, the denominator will remain low, resulting in a higher overall value. |
| $\text{IDN}=\sum_{i=1}^{N_{g}} \sum_{j=1}^{N_{g}} \frac{p(i,j)}{1+(\frac{\vert i-j\vert}{N_{g}})}$ | IDN (**inverse difference normalized**) is another measure of the local homogeneity of an image. Unlike Homogeneity1, IDN normalizes the difference between the neighboring intensity values by dividing over the total number of discrete intensity values. |
| $\text{Inverse variance}=\sum_{i=1}^{N_{g}} \sum_{j=1}^{N_{g}} \frac{p(i,j)}{\vert i-j\vert^{2}},i\neq j$ |  |
| $\text{Maximum probability}=max(p(i,j))$ | **Maximum Probability** is occurrences of the most predominant pair of neighboring intensity values (also known as Joint maximum). |
| $\text{Sum average}=\sum_{k=2}^{2N_{g}} p_{x+y}(k)k$ | **Sum Average** measures the relationship between occurrences of pairs with lower intensity values and occurrences of pairs with higher intensity values. |
| $\text{Sum entropy}=\sum_{k=2}^{2N_{g}} p_{x+y}(k)\log_{2}(p_{x+y}(k)+\epsilon)$ | **Sum Entropy** is a sum of neighborhood intensity value differences. |
| $\text{Sum squares}=\sum_{i=1}^{N_{g}} \sum_{j=1}^{N_{g}} (i-\mu_{x})^{2}p(i,j)$ | **Sum of Squares** or Variance is a measure in the distribution of neigboring intensity level pairs about the mean intensity level in the GLCM. (Defined by IBSI as Joint Variance). |
| Adapted from <http://pyradiomics.readthedocs.io/en/latest/features.html> – last accessed March 22, 2018. © Copyright 2016, pyradiomics community, http://github.com/radiomics/pyradiomics Revision eae15eff. | |

***d. Gray Level Size Zone Matrix (GLSZM)***

A Gray Level Size Zone (GLSZM) describes gray level zones in a ROI, which are defined as the number of connected voxels that share the same gray level intensity. A voxel is considered connected if the distance is 1 according to the infinity norm (26-connected region in a 3D, 8-connected region in 2D). In a gray level size zone matrix ***P(i,j)*** the **(i,j)^th^** element equals the number of zones with gray level ***i*** and size ***j*** appear in image. Contrary to GLCM and GLRLM, the GLSZM is rotation independent, with only one matrix calculated for all directions in the ROI (Table S9).

Let:

**N_g_** be the number of discreet intensity values in the image

**N_s_** be the number of discreet zone sizes in the image

**N_p_** be the number of voxels in the image

**N_z_** be the number of zones in the ROI, which is equal to $\sum_{i=1}^{N_{g}} \sum_{j=1}^{N_{s}} \text{P}(i,j)$ and ***1 ≤ N_z_ ≤ N_p_***

***P(i,j)*** be the size zone matrix

***p(i,j)*** be the normalized size zone matrix, defined as $p(i,j)=\frac{\text{P}(i,j)}{N_{z}}$

***ϵ*** is an arbitrarily small positive number (≈2.2×10−16).

| **Table S9. Gray Level Size Zone Matrix (GLSZM) statistics for PVAT characterization** | |
| --- | --- |
| **Radiomic feature** | **Interpretation** |
| $\text{SAE}=\frac{\sum_{i=1}^{N_{g}} \sum_{j=1}^{N_{s}} \frac{\text{P}(i,j)}{j^{2}}}{N_{z}}$ | **SAE (small area emphasis)** is a measure of the distribution of small size zones, with a greater value indicative of smaller size zones and more fine textures. |
| $\text{LAE}=\frac{\sum_{i=1}^{N_{g}} \sum_{j=1}^{N_{s}} \text{P}(i,j)j^{2}}{N_{z}}$ | **LAE (large area emphasis)** is a measure of the distribution of large area size zones, with a greater value indicative of larger size zones and more coarse textures. |
| $\text{GLN}=\frac{\sum_{i=1}^{N_{g}} {(\sum_{j=1}^{N_{s}} \text{P}(i,j))}^{2}}{N_{z}}$ | **GLN (gray level non-uniformity)** measures the variability of gray-level intensity values in the image, with a lower value indicating more homogeneity in intensity values. |
| $\text{GLNN}=\frac{\sum_{i=1}^{N_{g}} {(\sum_{j=1}^{N_{s}} \text{P}(i,j))}^{2}}{N_{z}^{2}}$ | **GLNN (gray level non-uniformity normalized)** measures the variability of gray-level intensity values in the image, with a lower value indicating a greater similarity in intensity values. This is the normalized version of the GLN formula. |
| $\text{SZN}=\frac{\sum_{j=1}^{N_{s}} {(\sum_{i=1}^{N_{g}} \text{P}(i,j))}^{2}}{N_{z}}$ | **SZN (size zone non-uniformity)** measures the variability of size zone volumes in the image, with a lower value indicating more homogeneity in size zone volumes. |
| $\text{SZNN}=\frac{\sum_{j=1}^{N_{s}} {(\sum_{i=1}^{N_{g}} \text{P}(i,j))}^{2}}{N_{z}^{2}}$ | **SZNN (size zone non-uniformity normalized)** measures the variability of size zone volumes throughout the image, with a lower value indicating more homogeneity among zone size volumes in the image. This is the normalized version of the SZN formula. |
| $\text{Zone Percentage}=\frac{N_{z}}{N_{p}}$ | **ZP (Zone Percentage)** measures the coarseness of the texture by taking the ratio of number of zones and number of voxels in the ROI. Values are in range $\frac{1}{N_{p}}\leq ZP\leq1$, with higher values indicating a larger portion of the ROI consists of small zones (indicates a more fine texture). |
| $\text{GLV}=\sum_{i=1}^{N_{g}} \sum_{j=1}^{N_{s}} p(i,j)(i-\mu)^{2}$, where $\mu=\sum_{i=1}^{N_{g}} \sum_{j=1}^{N_{s}} p(i,j)i$ | **Gray level variance (GLV)** measures the variance in gray level intensities for the zones. |
| $\text{ZV}=\sum_{i=1}^{N_{g}} \sum_{j=1}^{N_{s}} p(i,j)(j-\mu)^{2}$, where $\mu=\sum_{i=1}^{N_{g}} \sum_{j=1}^{N_{s}} p(i,j)j$ | **Zone Variance (ZV)** measures the variance in zone size volumes for the zones. |
| $\text{ZE}=-\sum_{i=1}^{N_{g}} \sum_{j=1}^{N_{s}} p(i,j)\log_{2}(p(i,j)+\epsilon)$ | **Zone Entropy (ZE)** measures the uncertainty/randomness in the distribution of zone sizes and gray levels. A higher value indicates more heterogeneneity in the texture patterns. |
| $\text{LGLZE}=\frac{\sum_{i=1}^{N_{g}} \sum_{j=1}^{N_{s}} \frac{\text{P}(i,j)}{i^{2}}}{N_{z}}$ | **LGLZE (low gray level zone emphasis)** measures the distribution of lower gray-level size zones, with a higher value indicating a greater proportion of lower gray-level values and size zones in the image. |
| $\text{HGLZE}=\frac{\sum_{i=1}^{N_{g}} \sum_{j=1}^{N_{s}} \text{P}(i,j)i^{2}}{N_{z}}$ | **HGLZE (high gray level zone emphasis)** measures the distribution of the higher gray-level values, with a higher value indicating a greater proportion of higher gray-level values and size zones in the image. |
| $\text{SALGLE}=\frac{\sum_{i=1}^{N_{g}} \sum_{j=1}^{N_{s}} \frac{\text{P}(i,j)}{i^{2}j^{2}}}{N_{z}}$ | **SALGLE (small area low gray level emphasis)** measures the proportion in the image of the joint distribution of smaller size zones with lower gray-level values. |
| $\text{SAHGLE}=\frac{\sum_{i=1}^{N_{g}} \sum_{j=1}^{N_{s}} \frac{\text{P}(i,j)i^{2}}{j^{2}}}{N_{z}}$ | **SAHGLE (small area high gray level emphasis)** measures the proportion in the image of the joint distribution of smaller size zones with higher gray-level values. |
| $\text{LALGLE}=\frac{\sum_{i=1}^{N_{g}} \sum_{j=1}^{N_{s}} \frac{\text{P}(i,j)j^{2}}{i^{2}}}{N_{z}}$ | **LALGLE (low area low gray level emphasis)** measures the proportion in the image of the joint distribution of larger size zones with lower gray-level values. |
| $\text{LAHGLE}=\frac{\sum_{i=1}^{N_{g}} \sum_{j=1}^{N_{s}} \text{P}(i,j)i^{2}j^{2}}{N_{z}}$ | **LAHGLE (low area high gray level emphasis)** measures the proportion in the image of the joint distribution of larger size zones with higher gray-level values. |
| Adapted from <http://pyradiomics.readthedocs.io/en/latest/features.html> – last accessed March 22, 2018. © Copyright 2016, pyradiomics community, http://github.com/radiomics/pyradiomics Revision eae15eff. | |

***e. Gray Level Run Length Matrix (GLRLM)***

A Gray Level Run Length Matrix (GLRLM) describes gray level runs, which are defined as the length in number of pixels, of consecutive pixels that have the same gray level value. In a gray level run length matrix ***P(i,j|θ)***, the ***(i,j)^th^*** element describes the number of runs with gray level ***i*** and length ***j*** occur in the image (ROI) along angle ***θ*** (Table S10).

Let:

***N_g_*** be the number of discreet intensity values in the image

***N_r_*** be the number of discreet run lengths in the image

***N_p_*** be the number of voxels in the image

***N_z_(θ)*** be the number of runs in the image along angle θ, which is equal to $\sum_{i=1}^{N_{g}} \sum_{j=1}^{N_{r}} \text{P}(i,j|\theta)$and 1≤N_z_(θ)≤N_p_

***P(i,j|θ)*** be the run length matrix for an arbitrary direction θ

***p(i,j|θ)*** be the normalized run length matrix, defined as $p(i,j|\theta)=\frac{\text{P}(i,j|\theta)}{N_{z}(\theta)}$

**ϵ** is an arbitrarily small positive number (≈2.2×10−16).

By default, the value of a feature is calculated on the GLRLM for each angle separately, after which the mean of these values is returned. If distance weighting is enabled, GLRLMs are weighted by the distance between neighbouring voxels and then summed and normalised. Features are then calculated on the resultant matrix. The distance between neighbouring voxels is calculated for each angle using the norm specified in ‘weightingNorm’

| **Table S10. Gray Level Run Length Matrix (GLRLM) statistics for PVAT characterization** | |
| --- | --- |
| **Radiomic feature** | **Interpretation** |
| $\text{SRE}=\frac{\sum_{i=1}^{N_{g}} \sum_{j=1}^{N_{r}} \frac{\text{P}(i,j\vert\theta)}{j^{2}}}{N_{z}(\theta)}$ | **SRE (Short Run Emphasis)** is a measure of the distribution of short run lengths, with a greater value indicative of shorter run lengths and more fine textural textures. |
| $\text{LRE}=\frac{\sum_{i=1}^{N_{g}} \sum_{j=1}^{N_{r}} \text{P}(i,j\vert\theta)j^{2}}{N_{z}(\theta)}$ | **LRE (Long Run Emphasis)** is a measure of the distribution of long run lengths, with a greater value indicative of longer run lengths and more coarse structural textures. |
| $\text{GLN}=\frac{\sum_{i=1}^{N_{g}} {(\sum_{j=1}^{N_{r}} \text{P}(i,j\vert\theta))}^{2}}{N_{z}(\theta)}$ | **GLN (Gray Level Non-uniformity)** measures the similarity of gray-level intensity values in the image, where a lower GLN value correlates with a greater similarity in intensity values. |
| $\text{GLNN}=\frac{\sum_{i=1}^{N_{g}} {(\sum_{j=1}^{N_{r}} \text{P}(i,j\vert\theta))}^{2}}{N_{z}(\theta)^{2}}$ | **GLNN (Gray Level Non-uniformity Normalized)** measures the similarity of gray-level intensity values in the image, where a lower GLNN value correlates with a greater similarity in intensity values. This is the normalized version of the GLN formula. |
| $\text{RLN}=\frac{\sum_{j=1}^{N_{r}} {(\sum_{i=1}^{N_{g}} \text{P}(i,j\vert\theta))}^{2}}{N_{z}(\theta)}$ | **RLN (Run Length Non-uniformity)** measures the similarity of run lengths throughout the image, with a lower value indicating more homogeneity among run lengths in the image. |
| $\text{RLNN}=\frac{\sum_{j=1}^{N_{r}} {(\sum_{i=1}^{N_{g}} \text{P}(i,j\vert\theta))}^{2}}{N_{z}(\theta)^{2}}$ | **RLNN (Run Length Non-uniformity)** measures the similarity of run lengths throughout the image, with a lower value indicating more homogeneity among run lengths in the image. This is the normalized version of the RLN formula. |
| $\text{RP}=\frac{N_{z}(\theta)}{N_{p}}$ | **RP (Run Percentage)** measures the coarseness of the texture by taking the ratio of number of runs and number of voxels in the ROI. Values are in range $\frac{1}{N_{p}}\leq RP\leq1$, with higher values indicating a larger portion of the ROI consists of short runs (indicates a more fine texture). |
| $\text{GLV}=\sum_{i=1}^{N_{g}} \sum_{j=1}^{N_{r}} p(i,j\vert\theta)(i-\mu)^{2}$,  where $\mu=\sum_{i=1}^{N_{g}} \sum_{j=1}^{N_{r}} p(i,j\vert\theta)i$ | **GLV (Gray Level Variance)** measures the variance in gray level intensity for the runs. |
| $\text{RV}=\sum_{i=1}^{N_{g}} \sum_{j=1}^{N_{r}} p(i,j\vert\theta)(j-\mu)^{2}$,  where $\mu=\sum_{i=1}^{N_{g}} \sum_{j=1}^{N_{r}} p(i,j\vert\theta)j$ | **RV (Run Variance)** is a measure of the variance in runs for the run lengths. |
| $\text{RE}=-\sum_{i=1}^{N_{g}} \sum_{j=1}^{N_{r}} p(i,j\vert\theta)\log_{2}(p(i,j\vert\theta)+\epsilon)$ | **RE (Run Entropy)** measures the uncertainty/randomness in the distribution of run lengths and gray levels. A higher value indicates more heterogeneity in the texture patterns. |
| $\text{LGLRE}=\frac{\sum_{i=1}^{N_{g}} \sum_{j=1}^{N_{r}} \frac{\text{P}(i,j\vert\theta)}{i^{2}}}{N_{z}(\theta)}$ | **LGLRE (low gray level run emphasis)** measures the distribution of low gray-level values, with a higher value indicating a greater concentration of low gray-level values in the image. |
| $\text{HGLRE}=\frac{\sum_{i=1}^{N_{g}} \sum_{j=1}^{N_{r}} \text{P}(i,j\vert\theta)i^{2}}{N_{z}(\theta)}$ | **HGLRE (high gray level run emphasis)** measures the distribution of the higher gray-level values, with a higher value indicating a greater concentration of high gray-level values in the image. |
| $\text{SRLGLE}=\frac{\sum_{i=1}^{N_{g}} \sum_{j=1}^{N_{r}} \frac{\text{P}(i,j\vert\theta)}{i^{2}j^{2}}}{N_{z}(\theta)}$ | **SRLGLE (short run low gray level emphasis)** measures the joint distribution of shorter run lengths with lower gray-level values. |
| $\text{SRHGLE}=\frac{\sum_{i=1}^{N_{g}} \sum_{j=1}^{N_{r}} \frac{\text{P}(i,j\vert\theta)i^{2}}{j^{2}}}{N_{z}(\theta)}$ | **SRHGLE (short run high gray level emphasis)** measures the joint distribution of shorter run lengths with higher gray-level values. |
| $\text{LRLGLRE}=\frac{\sum_{i=1}^{N_{g}} \sum_{j=1}^{N_{r}} \frac{\text{P}(i,j\vert\theta)j^{2}}{i^{2}}}{N_{z}(\theta)}$ | **LRLGLRE (long run low gray level emphasis)** measures the joint distribution of long run lengths with lower gray-level values. |
| $\text{LRHGLRE}=\frac{\sum_{i=1}^{N_{g}} \sum_{j=1}^{N_{r}} \text{P}(i,j\vert\theta)i^{2}j^{2}}{N_{z}(\theta)}$ | **LRHGLRE (long run high gray level run emphasis)** measures the joint distribution of long run lengths with higher gray-level values. |
| Adapted from <http://pyradiomics.readthedocs.io/en/latest/features.html> – last accessed March 22, 2018. © Copyright 2016, pyradiomics community, http://github.com/radiomics/pyradiomics Revision eae15eff. | |

***f. Neigbouring Gray Tone Difference Matrix (NGTDM) Features***

A Neighbouring Gray Tone Difference Matrix quantifies the difference between a gray value and the average gray value of its neighbours within distance ***δ*** (Table S11). The sum of absolute differences for gray level ***i*** is stored in the matrix. Let $\text{X}_{\mathbf{gl}}$be a set of segmented voxels and $x_{gl}(j_{x},j_{y},j_{z})\in\text{X}_{gl}$ be the gray level of a voxel at postion $(j_{x},j_{y},j_{z})$, then the average gray level of the neigbourhood is:

$$\begin{matrix} & \overline{A}_{i}=\overline{A}(j_{x},j_{y},j_{z}) \\ & =\frac{1}{W}\sum_{k_{x}=-\delta}^{\delta} \sum_{k_{y}=-\delta}^{\delta} \sum_{k_{z}=-\delta}^{\delta} x_{gl}(j_{x}+k_{x},j_{y}+k_{y},j_{z}+k_{z}), \\ & \text{where }(k_{x},k_{y},k_{z})\neq(0,0,0)\text{ and }x_{gl}(j_{x}+k_{x},j_{y}+k_{y},j_{z}+k_{z})\in\text{X}_{gl} \end{matrix}$$

Here, ***W*** is the number of voxels in the neighbourhood that are also in ***X_gl_***.

Let:

***n_i_*** be the number of voxels in ***X_gl_*** with gray level ***i***

***Nv,p*** be the total number of voxels in ***X_gl_*** and equal to $\sum n_{i}$ (i.e. the number of voxels with a valid region; at least 1 neighbor). $N_{v,p}\leq N_{p}$, where *N_p_* is the total number of voxels in the ROI.

***p_i_*** be the gray level probability and equal to $n_{i}/N_{v}$

$s_{i}=\{\begin{matrix} \sum^{n_{i}} \left| i-\overline{A}_{i} \right| & \text{for} & n_{i}\neq0 \\ 0 & \text{for} & n_{i}=0 \end{matrix}$ be the sum of absolute differences for gray level i

***N_g_*** be the number of discreet gray levels

***N_g,p_*** be the number of gray levels where ***p_i_***≠0

| **Table S11. Neigbouring Gray Tone Difference Matrix (NGTDM) for PVAT characterization** | |
| --- | --- |
| **Radiomic feature** | **Interpretation** |
| $\boldsymbol{Coarseness}=\frac{1}{\sum_{i=1}^{N_{g}} p_{i}s_{i}}$ | **Coarseness** is a measure of average difference between the center voxel and its neighbourhood and is an indication of the spatial rate of change. A higher value indicates a lower spatial change rate and a locally more uniform texture. |
| $\boldsymbol{Contrast}=\left( \frac{1}{N_{g,p}\left( N_{g,p}-1 \right)}\sum_{i=1}^{N_{g}} \sum_{j=1}^{N_{g}} p_{i}p_{j}(i-j)^{2} \right)\left( \frac{1}{N_{v,p}}\sum_{i=1}^{N_{g}} s_{i} \right)\text{, }$  $\text{where }p_{i}\neq0,p_{j}\neq0$ | **Contrast** is a measure of the spatial intensity change, but is also dependent on the overall gray level dynamic range. Contrast is high when both the dynamic range and the spatial change rate are high, i.e. an image with a large range of gray levels, with large changes between voxels and their neighbourhood. |
| $\boldsymbol{Busyness}=\frac{\sum_{i=1}^{N_{g}} p_{i}s_{i}}{\sum_{i=1}^{N_{g}} \sum_{j=1}^{N_{g}} \left\vert ip_{i}-jp_{j} \right\vert}\text{, }$  $\text{where }p_{i}\neq0,p_{j}\neq0$ | A measure of the change from a pixel to its neighbour. A high value for busyness indicates a ‘busy’ image, with rapid changes of intensity between pixels and its neighbourhood. |
| $\boldsymbol{Complexity}=\frac{1}{N_{v,p}}\sum_{i=1}^{N_{g}} \begin{aligned} \sum_{j=1}^{N_{g}} \left\vert i-j \right\vert\frac{p_{i}s_{i}+p_{j}s_{j}}{p_{i}+p_{j}}\text{, } \\ \end{aligned}$  $\text{where }p_{i}\neq0,p_{j}\neq0$ | An image is considered complex when there are many primitive components in the image, i.e. the image is non-uniform and there are many rapid changes in gray level intensity. |
| $\boldsymbol{Strength}=\frac{\sum_{i=1}^{N_{g}} \sum_{j=1}^{N_{g}} (p_{i}+p_{j})(i-j)^{2}}{\sum_{i=1}^{N_{g}} s_{i}}\text{, }$  $\text{where }p_{i}\neq0,p_{j}\neq0$ | **Strength** is a measure of the primitives in an image. Its value is high when the primitives are easily defined and visible, i.e. an image with slow change in intensity but more large coarse differences in gray level intensities. |
| Adapted from <http://pyradiomics.readthedocs.io/en/latest/features.html> – last accessed March 22, 2018. © Copyright 2016, pyradiomics community, http://github.com/radiomics/pyradiomics Revision eae15eff. | |

***g. Gray Level Dependence Matrix (GLDM)***

A Gray Level Dependence Matrix (GLDM) quantifies gray level dependencies in an image. A gray level dependency is defined as the number of connected voxels within distance ***δ*** that are dependent on the center voxel. A neighbouring voxel with gray level ***j*** is considered dependent on center voxel with gray level ***i*** if ***|i−j|≤α***. In a gray level dependence matrix ***P(i,j)*** the ***(i,j)^th^*** element describes the number of times a voxel with gray level ***i*** with ***j*** dependent voxels in its neighbourhood appears in image (Table S12).

***N_g_*** be the number of discreet intensity values in the image

***N_d_*** be the number of discreet dependency sizes in the image

***N_z_*** be the number of dependency zones in the image, which is equal to $\sum_{i=1}^{N_{g}} \sum_{j=1}^{N_{d}} \text{P}(i,j)$

***P(i,j)*** be the dependence matrix

***p(i,j)*** be the normalized dependence matrix, defined as $p(i,j)=\frac{\text{P}(i,j)}{N_{z}}$

| **Table S12. Gray Level Dependence Matrix (GLDM) statistics for PVAT characterization** | |
| --- | --- |
| **Radiomic feature** | **Interpretation** |
| $\boldsymbol{SDE}=\frac{\sum_{i=1}^{N_{g}} \sum_{j=1}^{N_{d}} \frac{\text{P}(i,j)}{i^{2}}}{N_{z}}$ | **SDE (Small Dependence Emphasis)**: A measure of the distribution of small dependencies, with a greater value indicative of smaller dependence and less homogeneous textures. |
| $\boldsymbol{LDE}=\frac{\sum_{i=1}^{N_{g}} \sum_{j=1}^{N_{d}} \text{P}(i,j)j^{2}}{N_{z}}$ | **LDE (Large Dependence Emphasis)**: A measure of the distribution of large dependencies, with a greater value indicative of larger dependence and more homogeneous textures. |
| $\boldsymbol{GLN}=\frac{\sum_{i=1}^{N_{g}} {(\sum_{j=1}^{N_{d}} \text{P}(i,j))}^{2}}{\sum_{i=1}^{N_{g}} \sum_{j=1}^{N_{d}} {\text{P}(i,j)}}$ | **GLN (Gray Level Non-Uniformity)**: Measures the similarity of gray-level intensity values in the image, where a lower GLN value correlates with a greater similarity in intensity values. |
| $\boldsymbol{DN}=\frac{\sum_{j=1}^{N_{d}} {(\sum_{i=1}^{N_{g}} \text{P}(i,j))}^{2}}{N_{z}}$ | **DN (Dependence Non-Uniformity)**: Measures the similarity of dependence throughout the image, with a lower value indicating more homogeneity among dependencies in the image. |
| $\boldsymbol{DNN}=\frac{\sum_{j=1}^{N_{d}} {(\sum_{i=1}^{N_{g}} \text{P}(i,j))}^{2}}{N_{z}^{2}}$ | **DNN (Dependence Non-Uniformity Normalized)**: Measures the similarity of dependence throughout the image, with a lower value indicating more homogeneity among dependencies in the image. This is the normalized version of the DLN formula. |
| $\boldsymbol{GLV}=\sum_{i=1}^{N_{g}} \sum_{j=1}^{N_{d}} p(i,j)(i-\mu)^{2}\text{, }$  $\text{where}\text{ }\mu=\sum_{i=1}^{N_{g}} \sum_{j=1}^{N_{d}} ip(i,j)$ | **GLV (Gray Level Variance)**: Measures the variance in grey level in the image. |
| $\boldsymbol{DV}=\sum_{i=1}^{N_{g}} \sum_{j=1}^{N_{d}} p(i,j)(j-\mu)^{2}\text{, }$  $\text{where}\text{ }\mu=\sum_{i=1}^{N_{g}} \sum_{j=1}^{N_{d}} jp(i,j)$ | **DV (Dependence Variance)**: Measures the variance in dependence size in the image. |
| $\boldsymbol{DE}=-\sum_{i=1}^{N_{g}} \sum_{j=1}^{N_{d}} p(i,j)\log_{2}(p(i,j)+\epsilon)$ | **DE (Dependence Entropy)**: Measures the entropy in dependence size in the image. |
| $\boldsymbol{LGLE}=\frac{\sum_{i=1}^{N_{g}} \sum_{j=1}^{N_{d}} \frac{\text{P}(i,j)}{i^{2}}}{N_{z}}$ | **LGLE (Low Gray Level Emphasis)**: Measures the distribution of low gray-level values, with a higher value indicating a greater concentration of low gray-level values in the image. |
| $\boldsymbol{HGLE}=\frac{\sum_{i=1}^{N_{g}} \sum_{j=1}^{N_{d}} \text{P}(i,j)i^{2}}{N_{z}}$ | **HGLE (High Gray Level Emphasis)**: Measures the distribution of the higher gray-level values, with a higher value indicating a greater concentration of high gray-level values in the image. |
| $\boldsymbol{SDLGLE}=\frac{\sum_{i=1}^{N_{g}} \sum_{j=1}^{N_{d}} \frac{\text{P}(i,j)}{i^{2}j^{2}}}{N_{z}}$ | **SDLGLE (Small Dependence Low Gray Level Emphasis)**: Measures the joint distribution of small dependence with lower gray-level values.C |
| $\boldsymbol{SDHGLE}=\frac{\sum_{i=1}^{N_{g}} \sum_{j=1}^{N_{d}} \frac{\text{P}(i,j)i^{2}}{j^{2}}}{N_{z}}$ | **SDHGLE (Small Dependence High Gray Level Emphasis)**: Measures the joint distribution of small dependence with higher gray-level values. |
| $\boldsymbol{LDLGLE}=\frac{\sum_{i=1}^{N_{g}} \sum_{j=1}^{N_{d}} \frac{\text{P}(i,j)j^{2}}{i^{2}}}{N_{z}}$ | **LDLGLE (Large Dependence Low Gray Level Emphasis)**: Measures the joint distribution of large dependence with lower gray-level values. |
| $\boldsymbol{LDHGLE}=\frac{\sum_{i=1}^{N_{g}} \sum_{j=1}^{N_{d}} \text{P}(i,j)i^{2}j^{2}}{N_{z}}$ | **LDHGLE (Large Dependence High Gray Level Emphasis)**: Measures the joint distribution of large dependence with higher gray-level values. |
| Adapted from <http://pyradiomics.readthedocs.io/en/latest/features.html> – last accessed March 22, 2018. © Copyright 2016, pyradiomics community, http://github.com/radiomics/pyradiomics Revision eae15eff. | |

**8. Supplemental Figures**


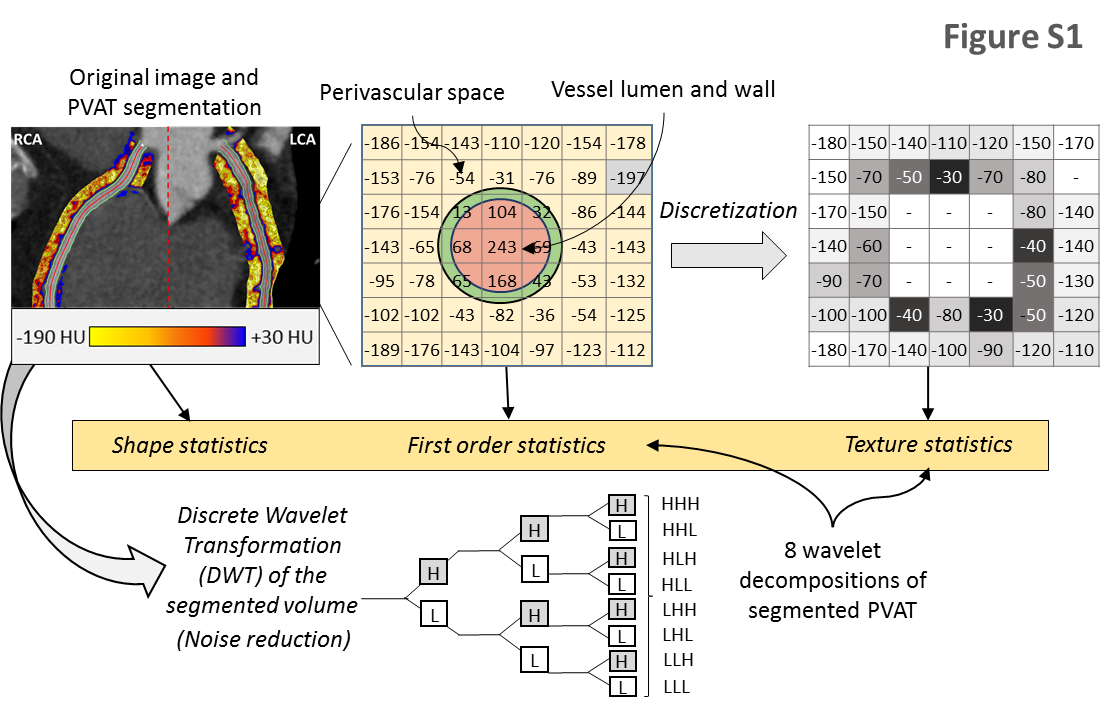


**Figure S1 | Radiomic analysis of perivascular adipose tissue (PVAT) around the coronary vessels.** The PVAT of the right (RCA) and left coronary arteries (LCA, left main and proximal to mid left anterior descending artery) was segmented and used to calculate a number of shape-, attenuation- and texture-related statistics. Attenuation values were discretized into 16 bins of equal 10 Hounsfield Unit (HU)-width to compute texture statistics, whereas additional wavelet transformations with a high- or low-filter were applied in the x, y and z plane. This yielded a total of 843 radiomic features for each territory, and a total of 1686 radiomic features for each patient and CCTA scan.

**
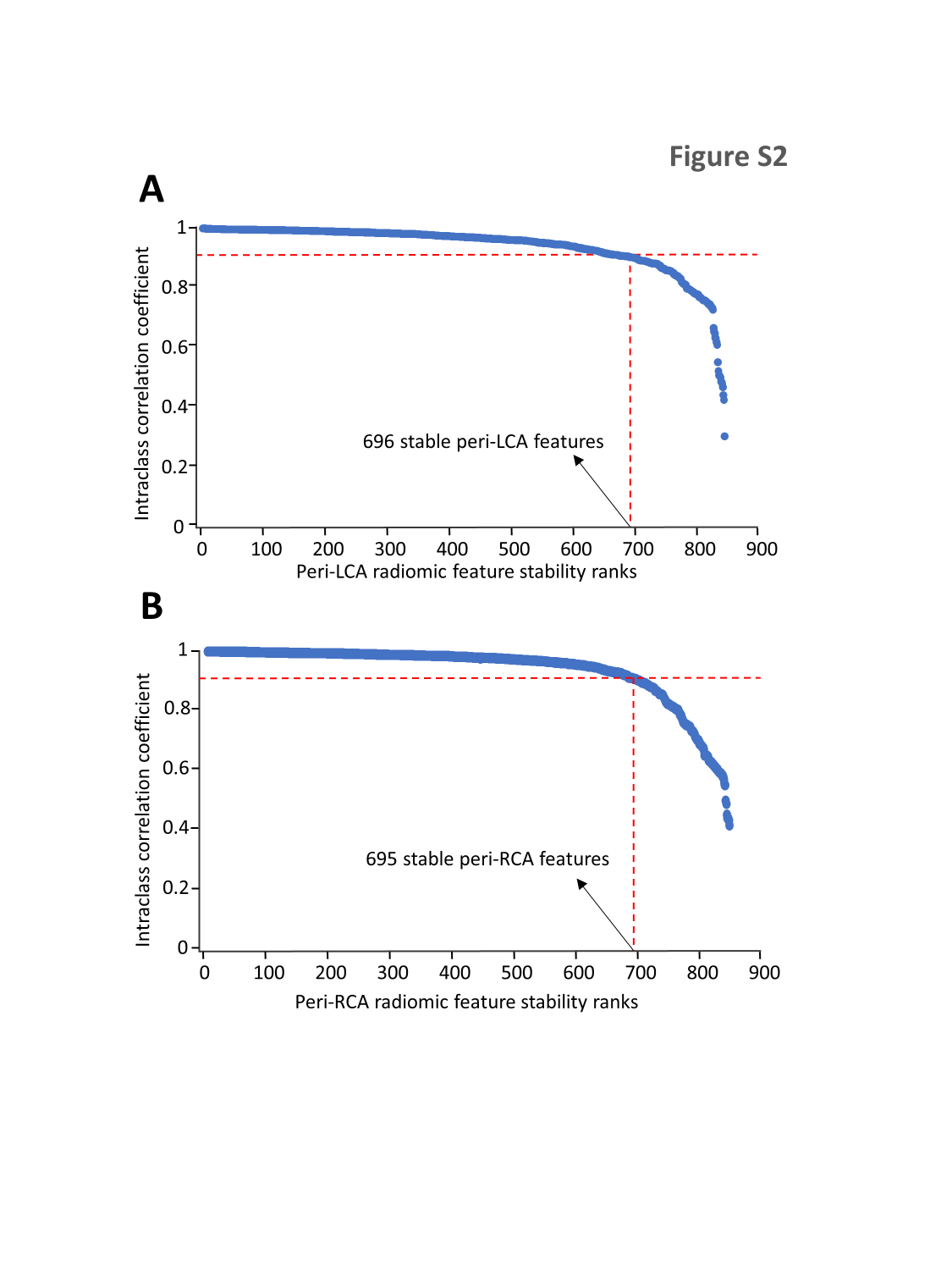
**

**Figure S2 | Stability analysis of coronary perivascular adipose tissue features.** Plot of the inter-observer intraclass correlation coefficient of all 843 radiomic features measured around the right **(A)** and left coronary arteries **(B)**. Radiomic features are ranked on descending order based on their ICC. A total of 696 and 695 radiomic features were found to have an ICC equal to or greater than 0.90 on inter-observer analysis around the RCA and LCA respectively and were subsequently selected for inclusion in further analyses. LCA: left coronary artery; RCA: right coronary artery.

**
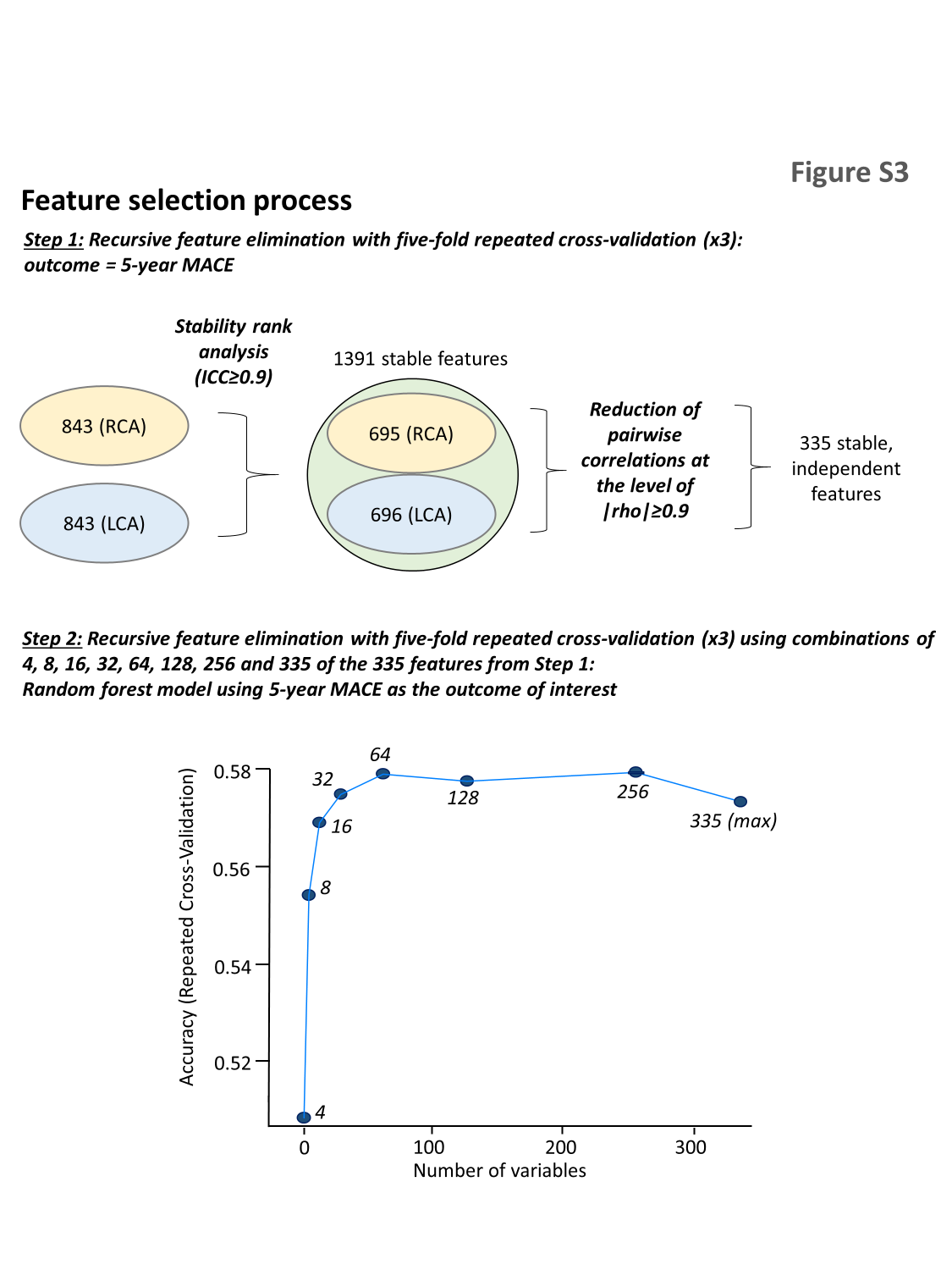
**

**Figure S3 | Radiomic feature selection process.** A total of 1391 out of 1686 (82.0%) features were found to be stable following intra-observer analysis, as defined by an intraclass correlation coefficient (ICC) of ≥0.9. Further reduction of pairwise correlations at the level of |rho|≥0.9 resulted in 335 independent features. Next, recursive feature elimination with a random forest algorithm and repeated five-fold cross-validation showed a plateau in the accuracy of the trained model with a maximal number of 64 selected features. LCA: left coronary artery; MACE: major adverse cardiac events; RCA: right coronary artery.

**
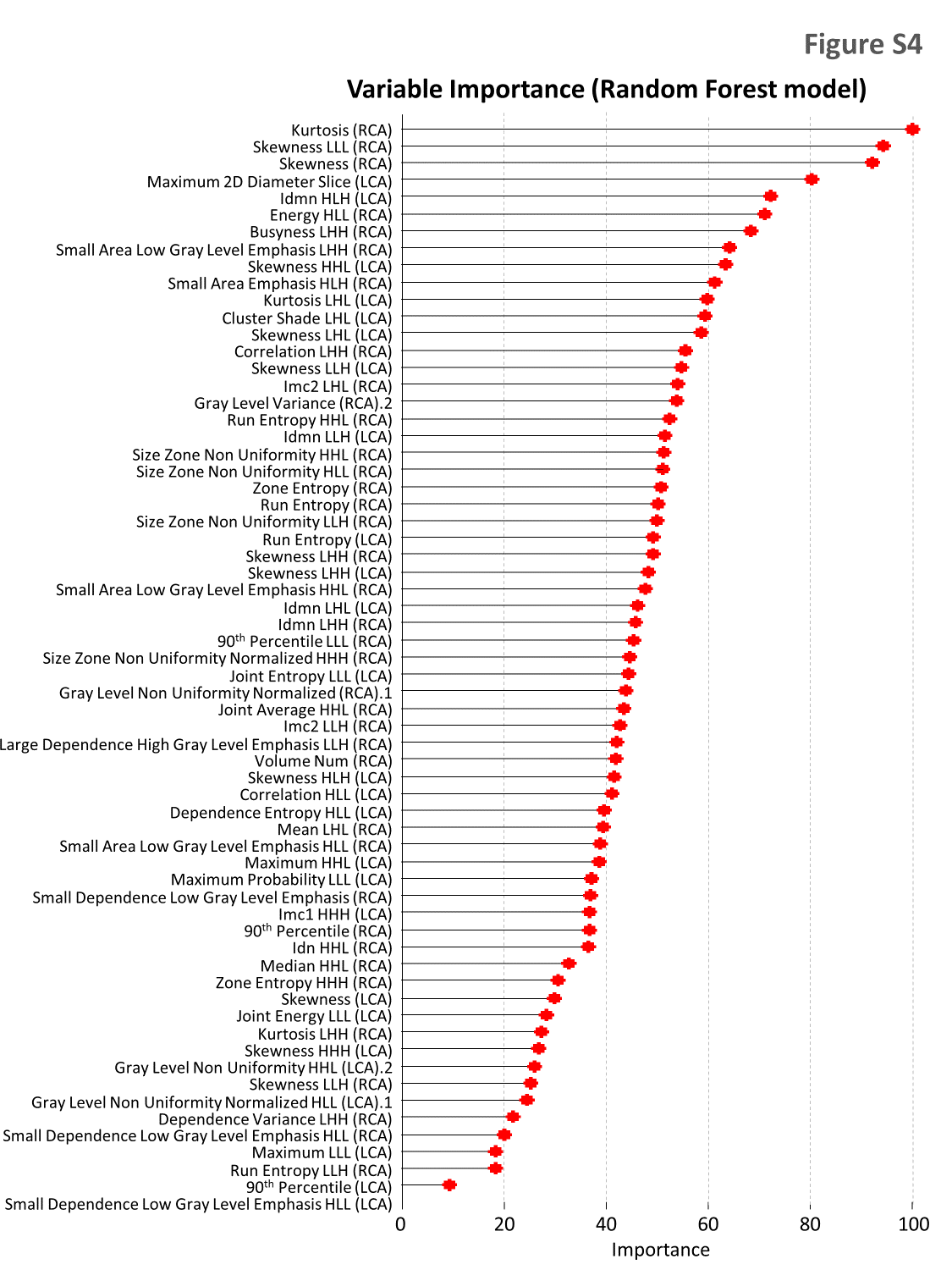
**

**Figure S4 | Variable importance in the final random forest model.** A graphical representation of the relative variable importance of the 64 selected radiomic features in the final random forest model for MACE discrimination. H: high wavelet filter; L: low wavelet filter; MACE: major adverse cardiac events.

**
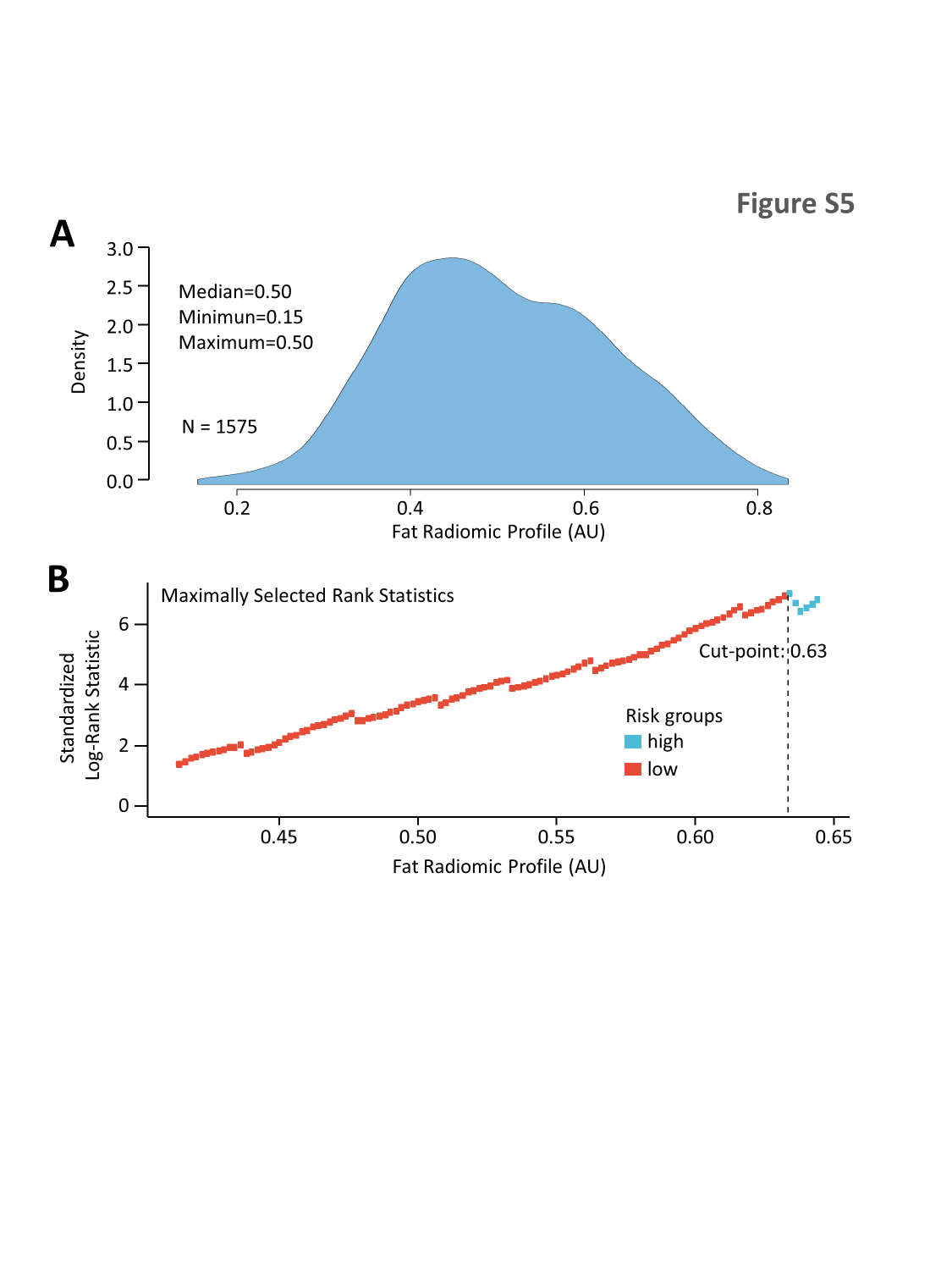
**

**Figure S5 | Identifying the optimal cut-off for the pericoronary fat radiomic profile (FRP). (A)** Density plot describing the distribution of FRP in the SCOT-HEART population (n=1575 individuals). **(B)** Plot of the standardized log-rank statistic for prediction of major adverse cardiac events versus different cut-off points for FRP, showing optimal discrimination for a cut-off point of 0.63. FRP: fat radiomic profile.

**9. Supplemental (online) References**

1. Antonopoulos AS, Sanna F, Sabharwal N, Thomas S, Oikonomou EK, Herdman L, Margaritis M, Shirodaria C, Kampoli AM, Akoumianakis I, Petrou M, Sayeed R, Krasopoulos G, Psarros C, Ciccone P, Brophy CM, Digby J, Kelion A, Uberoi R, Anthony S, Alexopoulos N, Tousoulis D, Achenbach S, Neubauer S, Channon KM, Antoniades C. Detecting human coronary inflammation by imaging perivascular fat. Sci Transl Med. 2017;**9**(398).

2. Oikonomou EK, Marwan M, Desai MY, Mancio J, Alashi A, Hutt Centeno E, Thomas S, Herdman L, Kotanidis CP, Thomas KE, Griffin BP, Flamm SD, Antonopoulos AS, Shirodaria C, Sabharwal N, Deanfield J, Neubauer S, Hopewell JC, Channon KM, Achenbach S, Antoniades C. Non-invasive detection of coronary inflammation using computed tomography and prediction of residual cardiovascular risk (the CRISP CT study): a post-hoc analysis of prospective outcome data. Lancet 2018;**392**(10151):929-939.

3. SCOT-HEART Investigators. CT coronary angiography in patients with suspected angina due to coronary heart disease (SCOT-HEART): an open-label, parallel-group, multicentre trial. Lancet 2015;**385**(9985):2383-91.

4. SCOT-HEART Investigators. Coronary CT Angiography and 5-Year Risk of Myocardial Infarction. N Engl J Med 2018;**379**(10):924-933.

5. van Griethuysen JJM, Fedorov A, Parmar C, Hosny A, Aucoin N, Narayan V, Beets-Tan RGH, Fillion-Robin JC, Pieper S, Aerts H. Computational Radiomics System to Decode the Radiographic Phenotype. Cancer Res 2017;**77**(21):e104-e107.

6. Leijenaar RT, Nalbantov G, Carvalho S, van Elmpt WJ, Troost EG, Boellaard R, Aerts HJ, Gillies RJ, Lambin P. The effect of SUV discretization in quantitative FDG-PET Radiomics: the need for standardized methodology in tumor texture analysis. Sci Rep 2015;**5**:11075.

7. Kolossvary M, Kellermayer M, Merkely B, Maurovich-Horvat P. Cardiac Computed Tomography Radiomics: A Comprehensive Review on Radiomic Techniques. J Thorac Imaging 2018;**33**(1):26-34.

8. Guo X, Liu X, Wang H, Liang Z, Wu W, He Q, Li K, Wang W. Enhanced CT images by the wavelet transform improving diagnostic accuracy of chest nodules. J Digit Imaging 2011;**24**(1):44-9.

9. Aerts HJWL, Velazquez ER, Leijenaar RTH, Parmar C, Grossmann P, Cavalho S, Bussink J, Monshouwer R, Haibe-Kains B, Rietveld D, Hoebers F, Rietbergen MM, Leemans CR, Dekker A, Quackenbush J, Gillies RJ, Lambin P. Decoding tumour phenotype by noninvasive imaging using a quantitative radiomics approach. Nature Communications 2014;**5**.

10. Agatston AS, Janowitz WR, Hildner FJ, Zusmer NR, Viamonte M, Jr., Detrano R. Quantification of coronary artery calcium using ultrafast computed tomography. J Am Coll Cardiol 1990;**15**(4):827-32.

11. Puchner SB, Liu T, Mayrhofer T, Truong QA, Lee H, Fleg JL, Nagurney JT, Udelson JE, Hoffmann U, Ferencik M. High-risk plaque detected on coronary CT angiography predicts acute coronary syndromes independent of significant stenosis in acute chest pain: results from the ROMICAT-II trial. J Am Coll Cardiol 2014;**64**(7):684-92.

12. Williams MC, Moss AJ, Dweck M, Adamson PD, Alam S, Hunter A, Shah ASV, Pawade T, Weir-McCall JR, Roditi G, van Beek EJR, Newby DE, Nicol ED. Coronary Artery Plaque Characteristics Associated With Adverse Outcomes in the SCOT-HEART Study. J Am Coll Cardiol 2019;**73**(3):291-301.

13. Antonopoulos AS, Margaritis M, Coutinho P, Shirodaria C, Psarros C, Herdman L, Sanna F, De Silva R, Petrou M, Sayeed R, Krasopoulos G, Lee R, Digby J, Reilly S, Bakogiannis C, Tousoulis D, Kessler B, Casadei B, Channon KM, Antoniades C. Adiponectin as a link between type 2 diabetes and vascular NADPH oxidase activity in the human arterial wall: the regulatory role of perivascular adipose tissue. Diabetes 2015;**64**(6):2207-19.

14. Margaritis M, Antonopoulos AS, Digby J, Lee R, Reilly S, Coutinho P, Shirodaria C, Sayeed R, Petrou M, De Silva R, Jalilzadeh S, Demosthenous M, Bakogiannis C, Tousoulis D, Stefanadis C, Choudhury RP, Casadei B, Channon KM, Antoniades C. Interactions between vascular wall and perivascular adipose tissue reveal novel roles for adiponectin in the regulation of endothelial nitric oxide synthase function in human vessels. Circulation 2013;**127**(22):2209-21.

15. Lee J. Patient-Specific Predictive Modeling Using Random Forests: An Observational Study for the Critically Ill. JMIR Med Inform 2017;**5**(1):e3.
